# FISHER'S GEOMETRIC MODEL WITH A MOVING OPTIMUM

**DOI:** 10.1111/evo.12465

**Published:** 2014-07-10

**Authors:** Sebastian Matuszewski, Joachim Hermisson, Michael Kopp

**Affiliations:** 1 Mathematics and BioSciences Group, Faculty of Mathematics, University of ViennaOskar-Morgenstern-Platz 1, A-1090, Vienna, Austria; 2 Max F. Perutz LaboratoriesDr.-Bohrgasse 9, A-1030, Vienna, Austria; 3 Aix Marseille Université, CNRS, Centrale Marseille, I2MUMR 7373, 13453, Marseille, France

**Keywords:** Adaptation, models/simulations, mutations, pleiotropy, population genetics, selection-natural

## Abstract

Fisher's geometric model has been widely used to study the effects of pleiotropy and organismic complexity on phenotypic adaptation. Here, we study a version of Fisher's model in which a population adapts to a gradually moving optimum. Key parameters are the rate of environmental change, the dimensionality of phenotype space, and the patterns of mutational and selectional correlations. We focus on the distribution of adaptive substitutions, that is, the multivariate distribution of the phenotypic effects of fixed beneficial mutations. Our main results are based on an “adaptive-walk approximation,” which is checked against individual-based simulations. We find that (1) the distribution of adaptive substitutions is strongly affected by the ecological dynamics and largely depends on a single composite parameter γ, which scales the rate of environmental change by the “adaptive potential” of the population; (2) the distribution of adaptive substitution reflects the shape of the fitness landscape if the environment changes slowly, whereas it mirrors the distribution of new mutations if the environment changes fast; (3) in contrast to classical models of adaptation assuming a constant optimum, with a moving optimum, more complex organisms evolve via larger adaptive steps.

Natural populations are constantly faced with environmental changes that force them to either adapt or go extinct. In *Arabidopsis thaliana*, Hancock et al. (2011) recently identified candidate single nucleotide polymorphisms scattered over the entire genome that affect flowering time and vernalization and are strongly correlated with climate variables. Likewise, annual cycles of reproduction of various plants and animals have been adjusted to the peak availability of food as a response to changing environments (Gienapp et al. 2013). Conversely, migratory bird species that fail to respond phenologically decline in population size (Møller et al. 2008). The brood parasitic common cuckoo (*Cuculus canorus*) population, for example, declined in size by 6% since 1980, as they failed to synchronize their reproductive and migratory cycles with those of their particular host species, to which they are adapted to in terms of egg size, coloration, and spottiness (Antonov et al. 2010; Møller et al. 2011).

In recent years, numerous theoretical studies of the population genetics of adaptation have attempted to provide a formal framework for the observed empirical phenomena (for a review see Orr 2005b). Central to these studies is the description of the fundamental event during adaptation, that is, the substitution of a resident allele (i.e., gene variant) by a beneficial mutation. The statistical description of this process has been at the heart of evolutionary biology (Charlesworth 1996), and is key to addressing seemingly simple questions, such as: From the set of mutations that emerge in a population, which are the ones that will get fixed and what is their effect on phenotype or fitness? Will adaptation proceed by many steps of small effect or just by a few adaptive substitutions of large effect? Do simple organisms evolve faster than complex ones?

One of the most influential models of adaptive phenotypic evolution is Fisher's geometric model (FGM) (Fisher 1930). In this model, a phenotype is treated as a point in a multidimensional trait space, and mutations are random vectors in this space, which are beneficial if they bring the mutant phenotype closer to a nearby local optimum. Thus, FGM implicitly assumes “universal pleiotropy” (each mutation affects every trait) and, therefore, equates pleiotropy with “organismic complexity.” Despite its simplicity and the lack of a clear genetic context (Chevin et al. 2010), FGM, more than 80 years after its proposal, has yielded several robust predictions supported by growing empirical evidence: First, the distribution of fitness effects of new mutations is well approximated by a (displaced) negative gamma distribution (Martin and Lenormand 2006a; for empirical support see Hietpas et al. 2013). Second, the distribution of adaptive substitutions is approximately exponential, meaning that most fixed mutations are of small and only a few are of large effect (Orr 1998; for empirical support see Rockman 2012, but see Bell 2009). Finally, fixed mutational effects become on average smaller as organismic complexity increases (Orr 2000; for empirical support see Cooper et al. 2007)—a phenomenon that has been termed “the cost of complexity” (Orr 2000; Welch and Waxman 2003; Wagner and Zhang 2011).

The classical version of FGM, however, only addresses the situation in which a population is confronted with constant stabilizing selection after a sudden change in the environment (e.g., Orr 2002; Martin and Lenormand 2006a). In nature, in contrast, environmental change may as often be gradual (Hairston et al. 2005; Thompson 2005; Parmesan 2006; Perron et al. 2008). Collins (2011b) recently emphasized that “using [models of] instantaneous environmental change to understand adaptive evolutionary responses to gradual change will not only underestimate the amount of adaptation, but also predict the wrong genotypic and phenotypic changes.” Indeed, the necessity to include gradual environmental change into studies of adaptive evolution has long been recognized in quantitative genetics (e.g., Maynard Smith 1976). A number of studies have focused on the so-called moving-optimum model, in which the optimal values of a quantitative trait change over time (Lynch and Lande 1993; Bürger and Lynch 1995; Waxman and Peck 1999; Bürger and Gimelfarb 2002; Nunney 2003; Collins et al. 2007; Gordo and Campos 2013); extensions include multivariate phenotypes and the effects of pleiotropic constraints (Jones et al. 2004; Gomulkiewicz and Houle 2009; Jones et al. 2012; Chevin 2013; Lourenço et al. 2013). The focus of these studies was, however, on the rate of adaptation (Lynch and Lande 1993; Bürger and Lynch 1995; Gomulkiewicz and Holt 1995; Nunney 2003; Hansen and Houle 2008; Chevin 2013; Kopp and Matuszewski 2014) and the evolution and maintenance of genetic variation (Bürger 1999; Waxman and Peck 1999; Bürger and Gimelfarb 2002; Jones et al. 2004, 2012; Gomulkiewicz and Houle 2009). In contrast, characteristics of individual substitutions have been addressed only recently (Collins et al. 2007; Kopp and Hermisson 2007, 2009a,b). In particular, Kopp and Hermisson (2007, 2009a) employed the moving-optimum model of a single quantitative trait to study the fixation time of single mutations and the order in which mutations of different phenotypic effect sizes become fixed. Their latest study (Kopp and Hermisson 2009b) addresses the distribution of adaptive substitutions during long-term adaptation. Specifically, they showed that this distribution is almost entirely determined by a scaled rate of environmental change γ, which combines ecological and genetic factors (see below), and is unimodal (with an intermediate mode) rather than exponential. That is, most substitutions have an intermediate phenotypic effect, while small- and large-effect substitutions are rare.

An obvious next question is how these results are affected if phenotypic adaptation to gradual change is constrained by pleiotropic correlations among the traits under selection (as frequently observed in nature; Svensson et al. 2001; Guerreiro et al. 2012; Roff and Fairbairn 2012). This is the aim of the present article. This way, we integrate two modeling traditions, which have had little overlap so far: on the one hand, the multivariate moving-optimum model as used by Jones et al. (2004, 2012), and on the other hand, Fisher's classical geometric model for the study of adaptive effect sizes (Fisher 1930; Orr 1998, 2000). We study how the expected distribution of adaptive steps is influenced by the rate of environmental change, the number of traits under selection (i.e., “organismic complexity”), and by selectional and mutational correlations (i.e., the shapes of the fitness landscape and the multivariate distribution of new mutations). Our analysis shows that the genetic basis of adaptation crucially depends on the tempo and mode of environmental change.

## Model and Methods

### MODEL DESCRIPTION

#### Phenotype, environmental change, and selection

We consider the evolution of *n* phenotypic traits **z** = (*z*_1_, …, *z*_*n*_)′, each of which is under Gaussian stabilizing selection with regard to a time-dependent optimum *z*_opt_(*t*):
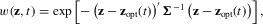
1where ′ denotes transposition and **Σ** (and thus also **Σ**^−1^) is an *n* × *n* positive definite and symmetric matrix. Throughout this article, we choose the linearly moving optimum,
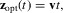
2where **v** = (*v*_1_, …, *v*_*n*_)′ is the vector of environmental change. In the following, we will interchangeably refer to *n* as the “degree of pleiotropy” or the “degree of complexity.”

The matrix **Σ** describes the shape of the fitness landscape (including a contribution of environmental noise to the phenotype **z**, which otherwise is not modeled explicitly; see Bürger 2000). We will say that selection is isotropic if **Σ** is proportional to an identity matrix, **Σ** = σ^2^**I** (σ^2^ > 0); and selection is correlated if **Σ** has nonzero off-diagonal entries. As a measure for the average width of the fitness landscape, we define
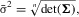
3which is the geometric mean of the eigenvalues of **Σ** (if the fitness landscape is represented by an ellipse, as in Figure S1_1 below, the axes of the ellipse have length proportional to the square root of the eigenvalues). Note that overall selection is strong if *δ*^2^ is small.

#### Genotypes and mutation

In accordance with Fisher's original model, we make the assumption of “universal pleiotropy,” that is, each mutation affects every trait. We denote by *α* the vector of the phenotypic effects of a mutation, and we assume that its distribution *p*(*α*) (which we will refer to as the distribution of new mutations) is multivariate normal with mean **0** and covariance matrix **M** (thus, we assume a continuum-of-alleles model), that is,
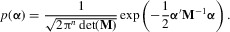
4Like **Σ**, **M** has dimensions *n* × *n* and must be symmetric and positive definite. The diagonal elements of **M** are the variances of the mutational effects for individual traits, whereas off-diagonal elements are the mutational covariances. We will say that mutation is isotropic if **M** is proportional to an identity matrix, **M** = *m*^2^**I** (*m*^2^ > 0), and mutation is correlated if **M** has nonzero off-diagonal entries. A measure for the average variance of mutational effects (in an arbitrary direction) is given by
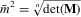
5

When comparing different degrees of pleiotropy/complexity, we typically assume that the distribution of mutational effects on a given trait is independent of the total number of traits *n* (so-called Euclidean superposition model; Turelli 1985; Wagner 1988; Wagner and Zhang 2011). For example, with isotropic mutation (see above), adding more traits does not change the parameter 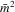. As a consequence, the average *total* effect of a mutation increases with *n*.

In Supporting Information 2, we introduce a transformation that shows that the general model outlined above can always be reduced to a model with isotropic selection (**Σ** = σ^2^**I**) and movement of the optimum along a single dimension (**v** = *v*_1_, 0, …, 0)′). In this transformed phenotype space, the effects of selectional and mutational correlations are entirely captured by the **M**-matrix (and, in particular, the orientation of its leading eigenvector/first principal component) relative to the direction of environmental change. Furthermore, all vectors (e.g., **z**, **v**, α) are measured relative to the average width of the distribution of new mutations 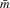.

### THE ADAPTIVE-WALK APPROXIMATION

The aim of this article is to investigate the distribution of adaptive substitutions ϕ(α), that is, the distribution of the effects of those mutations that eventually go to fixation and contribute to adaptation. Our main analytical tool will be the “adaptive-walk approximation.” Following Kopp and Hermisson (2009b), this approximation is based on the simplifying assumption that whether a new beneficial mutation goes to fixation or is lost by drift is determined immediately after its appearance and that, in the former case, fixation occurs instantaneously. Therefore, the population can be considered monomorphic nearly all of the time, and adaptation occurs as a series of discrete “steps,” which together will be referred to as an “adaptive walk” (Kauffman 1993; Orr 2000). This approximation ignores interactions between cosegregating mutations, such as epistasis, linkage, and Hill–Robertson interference (Hill and Robertson 1966).

Adaptive walks can easily be simulated using the following algorithm: (1) draw the waiting time for a new mutation from an exponential distribution with parameter Θ/2 (where Θ is a standard measure for the population- and genome-wide mutation rate); (2) draw the size of the mutation from its distribution *p*(α) (eq. 4); (3) accept the mutation (i.e., perform an adaptive step) with its fixation probability
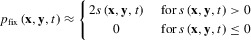
6(Haldane 1927), where **y** is the current population phenotype, **x** = **y** + α is the mutant phenotype, and
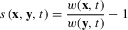
7denotes the selection coefficient of the mutant **x** in a wild-type population with phenotype **y** at time *t*. Note that equation 6 assumes *s* to be small and neglects chance fixations of deleterious mutations. In some simulations, we also used the slightly more accurate approximation *p*_fix_ ≈ max(0, 1 − exp(−2s)). (Even more accurate approximations exist that account for the change in the selection coefficient during the fixation process due to the environmental change, see Uecker and Hermisson 2011; however, within the simple framework of the adaptive-walk model we do not obtain further improvement.)

#### The distribution of adaptive substitutions

In the following we derive an analytical expression for the distribution ϕ(α|**y**) of the size α = **x** − **y** of the next adaptive substitution given an initial phenotype **y** at time *t* = 0. First, equation 7 can be approximated by
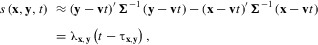
8awith
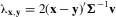
8b
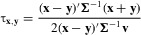
8c(provided *λ*_**x**,**y**_ ≠ 0). That is, with a linearly moving optimum, the selection coefficient increases or decreases approximately linearly over time, where *λ*_**x**,**y**_ is the rate of change and *τ*_**x**,**y**_ is the time when *s* reaches zero (the “lag time” in the terminology of Kopp and Hermisson 2007). This time dependence of the selection coefficient is illustrated in Supporting Information 1. The distribution ϕ(α|**y**) can then be calculated in four steps.

##### The instantaneous rate of substitutions

We denote by *g*(*t*, **y**) the rate at which substitutions of any kind happen at time *t*. *g*(*t*, **y**) is given by
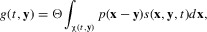
9where *χ* (*t*, **y**) = {**x**| *s*(**x**, **y**, *t*) > 0} is the set of all mutant phenotypes with positive selection coefficient at time *t*. The integrand in 11 is simply the product of the probability that a mutation with phenotype **x** arises (Θ*p*(α)/2) and its probability of fixation approximated as (2*s*(**x**, **y**, *t*), see eq. 6).

#### The waiting-time distribution

We denote by *F*(*t*|**y**) the probability that no fixation has happened before time *t* (thus, 1 − *F*(*t*|**y**) is the cumulative distribution function of the waiting time to the next fixation). From the theory of Poisson processes,
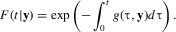
10

#### The conditional distribution of step sizes

The distribution of step sizes, given that the step occurs at time *t*, is simply proportional to the distribution of new mutations weighted by the selection coefficient (Gillespie 1983; Kopp and Hermisson 2009b):
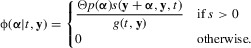
11

#### The distribution of step sizes

Finally, the unconditional distribution of the size of the next adaptive step can be calculated by integrating over all possible waiting times (see Kopp and Hermisson 2009b), yielding
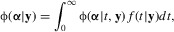
12awhere *f*(*t*|**y**) = (1 − *F*(*t*|**y**))′ is the density of the waiting-time distribution. Equation 14 can also be written as
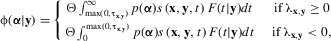
12bwhere *τ*_**x**,**y**_ is given by equation 10.

#### The parameter γ

Supporting Information 3 shows that the distribution of step sizes in the adaptive-walk approximation depends only on the distribution of new mutations and the composite parameter
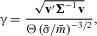
13where the term in the numerator can be interpreted as the rate of environmental change relative to the width of the fitness landscape in the direction of the moving optimum. If selection is isotropic, equation 16 reduces to
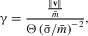
14which is equivalent to the γ defined by Kopp and Hermisson (2009b) for the single-trait case, except for differences in notation, and is independent of *n*. Here, 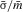 describes the mean width of the fitness landscape relative to the mean effect size of new mutations, and 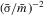 can be seen as a scale-free measure for the strength of stabilizing selection. The product of this term and the population-wide mutation rate Θ determines the “adaptive potential” of the population, γ can, thus, be interpreted as a scaled rate of environmental change (how fast the population needs to adapt relative to how readily it can adapt). In particular, it can be used to distinguish two limiting cases (Kopp and Hermisson 2009b). If γ is small, the population can easily follow the optimum. The adaptive process is, therefore, *environmentally limited*, and the distribution of adaptive substitutions is primarily determined by the lag time *τ*_**x**,**y**_, which determines when a mutation of effect α **x** − **y** becomes beneficial (“dynamic sieve” sensu Kopp and Hermisson 2009b). If γ is large, the population will follow the optimum with a large lag. In this case, the adaptive process is *genetically limited*, and the distribution of adaptive substitutions is largely determined by the distribution of new mutations *p*(α) (“static sieve” sensu Kopp and Hermisson 2009b). Numerical values for γ in these two regimes are discussed in Supporting Information 3.

#### The environmentally limited regime

In the environmentally limited regime, the Gaussian distribution of new mutations, *p*(α), can be approximated by a uniform distribution *p*_*u*_(**α**) with the same density at **α** = 0, that is,
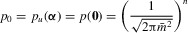
15(see Kopp and Hermisson 2009b). This approximation is justified if the optimum moves so slowly that all beneficial mutations are small (α close to **0**). It allows to directly calculate several properties of the distribution of adaptive substitutions. In particular, if the wild-type phenotype **y** = 0 (i.e., the population is perfectly adapted at time *t* = 0), the distribution of the “first” substitution (and all its moments) can be calculated analytically (Supporting Information 4).

### INDIVIDUAL-BASED SIMULATIONS

In addition to our adaptive-walk approximation, we conducted individual-based simulations (implemented in C++, available at doi:10.5061/dryad.534f0; see Bürger 2000; Kopp and Hermisson 2009b), which allow multiple mutations to segregate simultaneously, while making additional assumptions about the genetic architecture of the selected traits, the life cycle of individuals and the regulation of population size.

The simulations follow the evolution of a population of individuals with discrete and nonoverlapping generations. Individuals are characterized by *L* unlinked diploid loci, which additively determine the *n*-dimensional phenotype **z**. According to the universal-pleiotropy assumption, each allele at each locus is specified by a vector of contributions to the *n* traits. We neglect environmental variance and, therefore, equate genotypic and phenotypic values. Mutations occur at rate *u* per (diploid) locus and have effects drawn from the distribution *p*(α) (eq. 4). Each generation, the following steps are performed:

(1) *Viability selection*: Individuals are removed with probability 1 − *w*(**z**) (eq. 1).
(2) *Population regulation*: If, after selection, the population size *N* exceeds the carrying capacity *K*, *N* − *K* randomly chosen individuals are killed (Bürger 2000).
(3) *Reproduction*: The surviving individuals are randomly assigned to mating pairs, and each mating pair produces exactly *B* offspring (typically, *B* = 4). Note that, with this procedure, the effective size of a well-adapted population exceeds the census size (e.g., for *B* = 4, *N*_*e*_ = 4/3 *N*; Bürger 2000, p. 274). The offspring genotypes are derived from the parent genotypes by taking into account segregation, recombination, and mutation.

To monitor adaptive substitutions, the program keeps track of the genealogical relationship between the alleles at a given locus. A substitution is recorded whenever there is a change in the root of such an “allele tree” (i.e., when the surviving alleles get a new most recent common ancestor). This is equivalent to calling an allele fixed if the entire population has been taken over by that allele or its descendants (e.g., Gillespie 1993; Park and Krug 2007).

In all simulations, the initial population contained *K* = 1000 identical, homozygous individuals with phenotype **0** (i.e., the population was perfectly adapted at time 0). The number of loci was set to *L* = 10 and the mutation rate per diploid locus to *μ* = 5 × 10^−6^ per generation. This yields a population- and genome-wide mutation rate Θ = 2*NL*_*u*_ = 0.2. We chose this value to limit complications from interference between alleles cosegregating at the same locus, which have been thoroughly studied for the one-trait case in Kopp and Hermisson (2009b) (see Discussion). When comparing individual-based simulations to adaptive-walk simulations with differing Θ, the speed of environmental change **v** was adjusted accordingly to reach the same value of γ (eq. 16). Simulations were stopped after 1000 substitutions had been recorded. Alternatively, we only recorded the first adaptive substitution for 1000 replicate runs to study the initial phase of the adaptive process. Finally, for some parameter combinations, the simulations terminated because the population went extinct (e.g., if the environmental change was too fast).

## Results

Our primary interest is the distribution of adaptive substitutions, that is, the distribution ϕ(α) of the effects of those mutations that go to fixation and contribute to adaptation (eq. 12).

### THE DISTRIBUTION OF ADAPTIVE SUBSTITUTIONS AND PHENOTYPIC COMPLEXITY

Key properties of the distribution of adaptive substitutions can already be seen from a simplified model in which both mutation and selection are isotropic. Note that any model in which the two matrices **M** and Σ are proportional to each other, that is, have the same shape and orientation, can be reduced to this case via the transformation described in Supporting Information 2. The same holds true for any model under the environmentally limited regime, in which the shape of the **M**-matrix is irrelevant (because any distribution of new mutations can be approximated by a uniform distribution).

**Figure 1:**
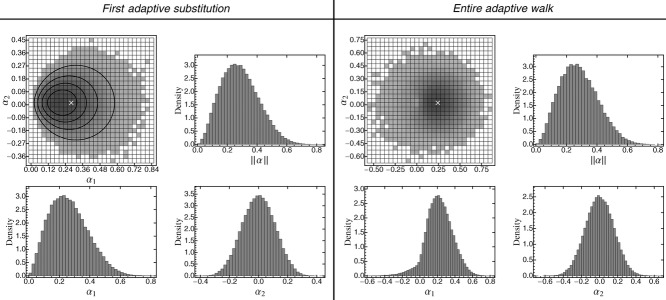
The multivariate distribution of the first adaptive substitution (left) and for the entire adaptive walk (right) for *n* = 2 traits, when the optimum moves slowly in the direction of the first trait. In the top-left figures on each side, shades of gray indicate the frequency of a given step size in adaptive-walk simulations with normally distributed mutational effects (with dark gray corresponding to high frequency), with the white cross showing the observed mean. The contour lines on the left represent the probability density predicted for a uniform distribution of new mutations (environmentally limited regime, eq. S19; highest probability density intervals for 0.25, 0.5, 0.75, 0.95 from inside out). Histograms show the marginal distribution of the first and second trait, α_1_ and α_2_, and the distribution of the total step size ||*α*||. Parameter values are *v*_1_ = 10^−5^, Θ = 1, σ^2^ = 10, ρΣ = 0, *m*^2^ = 1 ρM = 0 the scaled rate of environmental change *γ* = 10^−4^.

In the isotropic model, the distribution of adaptive substitutions is symmetric around the direction of the moving optimum. Figure 1 shows this distribution in adaptive-walk simulations with *n* = 2 traits. The marginal distribution in the direction of the optimum has an intermediate mode and resembles a gamma distribution, in accordance with previous results for the one-dimensional moving-optimum model (Kopp and Hermisson 2009b), and in contrast to the exponential pattern predicted for the classical Fisher model with constant selection (Orr 1998). Although the population always follows the optimum, pleiotropic side effects of fixed mutations frequently lead to maladaptation of the traits under pure stabilizing selection. The distribution of these deviations is bell-shaped and centered around zero (Fig. 1).

For small γ, explicit analytical results can be obtained for the distribution of the first adaptive substitution in the environmentally limited regime (Supporting Information 4, eq. S19). In particular, assuming **v** = (*v*_1_, 0, … 0), the mean and variance in the direction of the optimum are given by
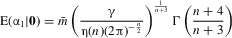
16
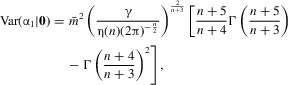
17where 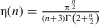 and 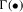 denotes the gamma function. Interestingly, the coefficient of variation 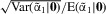 depends only on *n* (see Fig. S4_2). The variance in directions orthogonal to the optimum is given by
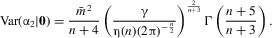
18

Additional results regarding higher moments of α_1_ and α_2_, the total step size ||*α*|| and the magnitude of pleiotropic deviations are given in Supporting Information 4.

**Figure 2:**
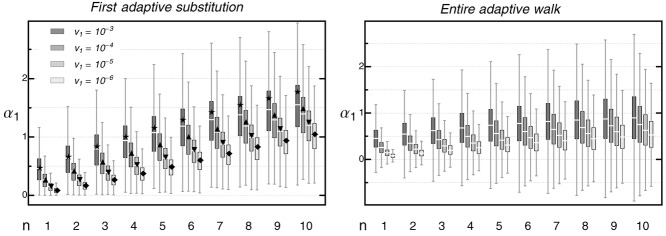
Distribution of the size α_1_ of the first adaptive substitution (left) and for the entire adaptive walk (right) in the direction of the moving optimum, as a function of phenotypic complexity *n* for different rates of environmental change *v*_1_. Symbols in the left-hand panel show the predicted mean of the first adaptive substitution when assuming a uniform distribution of mutational effects (environmentally limited regime, eq. 19). This approximation produces a good match as long as the predicted 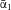 does not exceed the (mean) standard deviation of the effects of new mutations (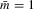). Beyond this mark, the realized step size is reduced due to limited availability of large-effect mutations. Compared to the first step, the increase of 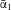 with *n* is less pronounced when considering the entire adaptive walk. The reason is that subsequent substitutions will often compensate for pleiotropic side effects of previous steps rather than follow the moving optimum. Boxplots are based on 10, 000 replicated adaptive-walk simulations. The box contains 50% of the data. Horizontal white bars indicate the mean step size 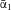. Whiskers extend to maximally 1.5 times the size of the box. Outliers are not shown. Parameters: σ^2^ = 10, ρΣ = 0, Θ = 1, *m*^2^ = 1, ρ*M* = 0; the scaled rate of environmental parameter *γ* = 10 × *v*_1_.

In accordance with previous findings (Collins et al. 2007; Kopp and Hermisson 2007, 2009a, b), equations 19–21 show that the mean step size in the direction of the optimum increases with the scaled rate of environmental change γ, and so does the magnitude of pleiotropic deviations (Figs. S4_1, S5_1). This fundamental relationship also holds true over the entire adaptive walk and beyond the environmental limit (Figs.2, S5_2, S5_3).

Some discussion is warranted regarding the dependence of the mean step size on the average variance of mutational effects 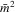. Increasing 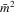 decreases γ and, consequently, leads to a reduced mean step size in the transformed phenotype space (see Supporting Information 2, eq. S28), where phenotypes are measured relative to 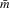. When phenotypes are measured in arbitrary units, however, the mean step size increases with 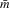 (eq. 19, see also Kopp and Hermisson 2009b). The reason is that an increase in 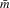 reduces the rate of appearance of small mutations (and, hence the parameter *p*_0_ in the environmental limit, see eq. 18), which reduces the ability of the population to follow the optimum closely.

A key result of our analysis is that, for a given speed of environmental change, the mean step size in direction of the optimum increases with the number of traits under selection, that is, with the level of pleiotropy or organismic complexity (eq. 19, Figs.2, S4_1, see also Figs. S5_2 and S5_3), and a similar result also holds for fitness (Fig. S5_4). At first, this result seems to contradict the “cost of complexity” argument from Fisher's model, which states that, in complex organisms, large mutations are unlikely to contribute to adaptation. The explanation is that, precisely because fewer mutations are beneficial if *n* is large (because there are more directions in which they can “go wrong”; Orr 1998), the time to the first step increases (see Supporting Information 4). By this time, the optimum has already moved considerably, such that also large mutations are beneficial (see Supporting Information 1 and Fig. S1_1), even if they have significant pleiotropic effects. These effects—in particular, the increased waiting time between adaptive substitutions—also affect population persistence: as shown in Figure S5_5, the mean time to extinction decreases with organismic complexity, and so does the maximal rate of environmental change a population can tolerate (Bürger and Lynch 1995).

### SELECTIONAL AND MUTATIONAL CORRELATIONS

**Figure 3:**
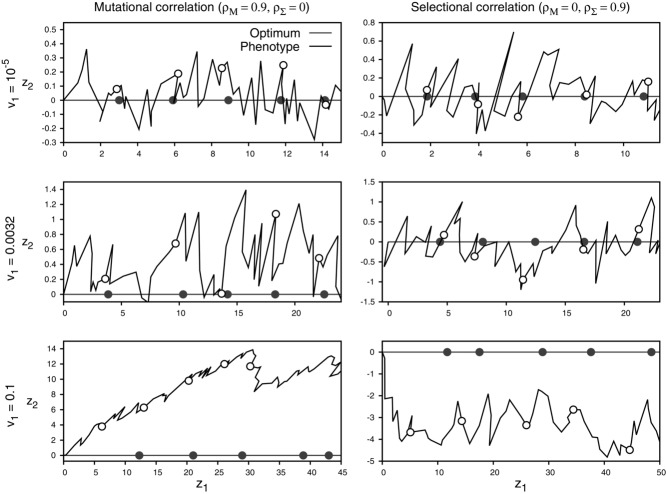
Example trajectories of the mean phenotype 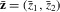 from adaptive-walk simulations with *n* = 2 traits and strong mutational or selectional correlation for three different rates of environmental change *v*_1_ (Fig. 4). Open circles mark the state of the population after 10, 20, 30, 40, and 50 adaptive substitutions. Closed circles give the corresponding positions of the moving optimum. The bottom row illustrates the flying- and diving-kite effect, respectively. Other parameters: Θ = 1, Σ^2^, *m* ?= 1.

To study the orientation of the distribution of step sizes in the *n*-dimensional phenotype space, we now consider a model with correlated selection and correlated mutations. We will assume that the angle between the direction of the optimum **v** and the leading eigenvector of Σ and/or **M** is 45°. More precisely, the optimum moves along the first trait axis (**v** = (v_1_, 0, …)′), whereas the fitness landscape and/or the distribution of new mutations are concentrated along the main diagonal: All diagonal elements of Σ (**M**) are equal to σ^2^ (*m*^2^) and all off-diagonal elements have magnitude ρΣσ^2^(ρ_*M*_*m*^2^), where 1 > ρΣ ≥ 0 (1 > ρ_*M*_ ≥ 0) is the magnitude of selectional (mutational) correlation. In this case, the fitness landscape (distribution of new mutations) is symmetric around the leading eigenvector of **Σ**(**M**), **ν** = (1, 1, 1, …). We first study the effects of mutational and selectional correlation separately. Exemplary adaptive walks for strong correlations are shown in Figure 3.

**Figure 4:**
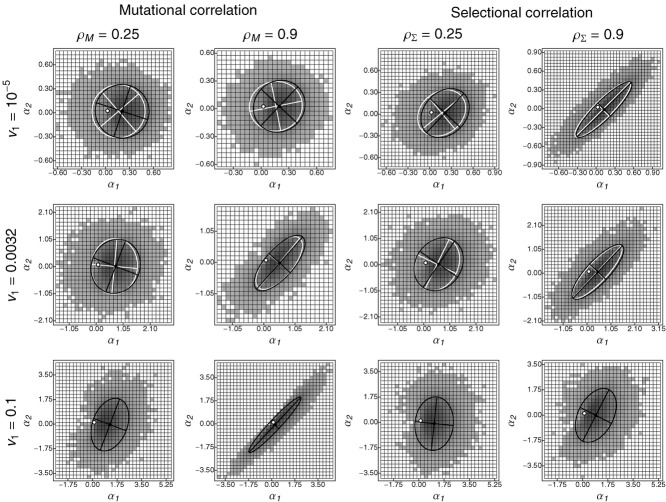
The distribution of adaptive substitutions for *n* = 2 traits under mutational or selectional correlation and their dependence on the speed of environmental change *v*_1_. Shades of gray indicate the frequency of a given step size in adaptive-walk simulations, and dark ellipses are the corresponding 90% confidence ellipses (based on the empirical covariance matrix). Light ellipses are 90% confidence ellipses for the step-size distribution from individual-based simulations (absent for *v*_1_ = 0.1 because simulated populations went extinct). The white dots mark the origins of the coordinate systems. Columns 1 and 2 are for weak and strong mutational correlations, respectively, with uncorrelated selection (ρΣ = 0). Columns 3 and 4 show results for selectional but no mutational correlation (ρ*M* = 0). Remaining parameters: Θ = 1, σ^2^ = 10, *m*^2^ = 1.

**Figure 5:**
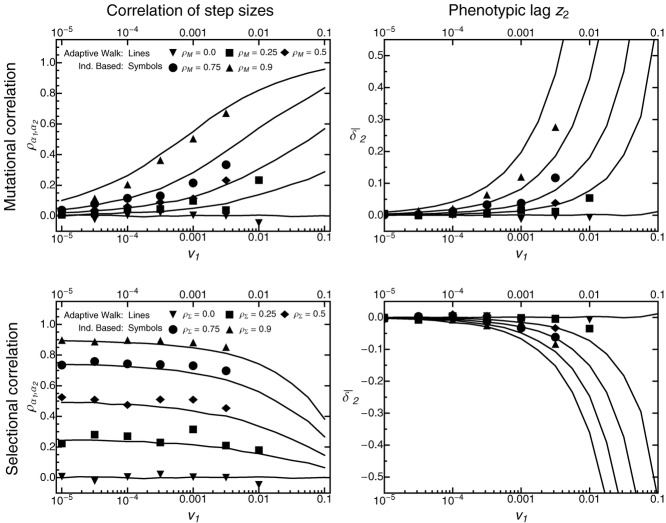
The impact of mutational and selectional correlations on the distribution of adaptive substitutions for *n* = 2 traits. The left-hand column shows the correlation 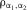 between step sizes in the direction of the moving optimum (α_1_) and in an orthogonal direction (α_2_) for different values of mutational (top row) and selectional (bottom row) correlation ρ_Σ_ and ρ_*M*_, plotted as a function of the rate of environmental change *v*_1_. The right-hand column shows 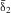, that is, the mean phenotypic lag in the direction orthogonal to the moving optimum, demonstrating the flying- and diving-kite effects (top and bottom, respectively). Lines show results from adaptive-walk simulations, whereas symbols are from individual-based simulations. Remaining parameters: Θ = 1, σ^2^ = 10, *m*^2^ = 1.

Figure 4 shows the multivariate distribution of adaptive substitutions, ϕ(α), for different strengths of selectional and mutational correlations under varying speeds of environmental change for *n* = 2 traits. As in the isotropic case (Fig. 1), the distribution ϕ(α) is biased toward the direction of the optimum, with pleiotropic side effects of fixed mutations on average being neutral (Figs. S5_6, S5_7). The shape of the distribution, however, critically depends on the interaction between the type and strength of correlations and the rate of environmental change. Mutational correlations tend to align the distribution of adaptive substitutions along the leading eigenvector of **M**, with stronger mutational correlations leading to stronger correlation in step sizes (Figs.4, 5, and S5_8 top left). This effect is strongest in fast-changing environments and gradually gets weaker as the rate of environmental change decreases (Fig. 5), until becoming almost unnoticeable. Selectional correlations similarly orientate the distribution of adaptive substitutions along to the leading eigenvector of Σ (Figs.4, 5 bottom left, S5_8). In contrast to mutational correlations, however, their impact is strongest if environmental change is slow (for small γ and the first step, the correlation is given by 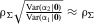, see eq. S36). Correlations in step sizes remain almost unchanged for a broad range of rates *v*_1_, before dropping off sharply once environmental change gets sufficiently fast.

**Figure 6:**
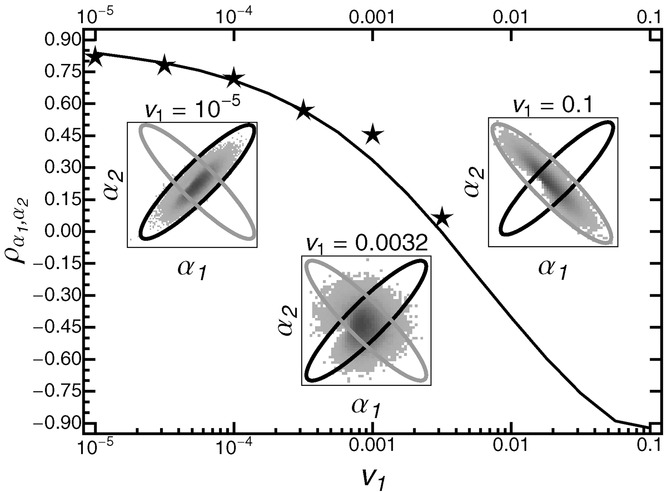
Correlation between steps in direction of the moving optimum and in a direction orthogonal to the moving optimum, 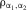, as function of the rate of environmental change *v*_1_, for strong and antagonistic mutational and selection correlation. The black line shows results from adaptive-walk simulations, while the stars give the corresponding individual-based simulation results. Insets give the distribution of adaptive substitutions retrieved from the adaptive-walk simulations for *v*_1_ = 10^−5^ *v*_1_ = 0.0032, and *v* = 0.1, with shades of gray indicating the frequency of a specific step size (dark gray indicating high frequency). The black and gray ellipses show the shape of the fitness landscape and the shape of the distribution of new mutations, respectively. Note that individual-based simulations died out for *v*_1_ > 0.0032. Remaining parameters: Θ = 1, σ^2^ = 10, ρ_Σ_ = 0.9, *m*^2^ = 1, ρ*M* = −0.9

These results still hold true when mutational and selectional correlations are both present but with opposite signs. As shown in Figure 6, the correlations in step sizes resemble the selectional correlations if environmental change is slow and resemble the mutational correlations if environmental change is fast. At intermediate rates of environmental change, the two effects cancel, and correlations in step sizes are close to zero.

Mutational and selectional correlations depend on the coordinate system in which multivariate phenotypes are measured (i.e., on the definition of traits). As shown in Supporting Information 2, there is always a transformation to coordinates in which selection (but not necessarily mutation) is isotropic. The key question, therefore, is whether the distribution of new mutations is aligned with the fitness landscape (in terms of the eigensystems/principle components of the matrices **M** and Σ). Our results can, thus, be reformulated as follows: The distribution of adaptive substitutions reflects the shape and orientation of the fitness landscape if adaptation is environmentally limited (i.e., if the optimum changes slowly), whereas it mirrors the distribution of new mutations (but with a mean shifted in the direction of the optimum) if adaptation is genetically limited (i.e., if the environment changes fast). Intuitively, as long as environmental change is slow, the population is close to the optimum and the shape of the distribution of new mutations is practically irrelevant, because only a small subset of new mutations from the center of their distribution can pass the selective sieve (Kopp and Hermisson 2009b). In contrast, if environmental change is fast, the population is far from the optimum, and the selective sieve has less impact on the adaptive process than the supply with new mutations. In the limit, pleiotropic side effects become negligible and the selection coefficient of new mutations depends only on their effect in the direction of the optimum.

**Figure 7:**
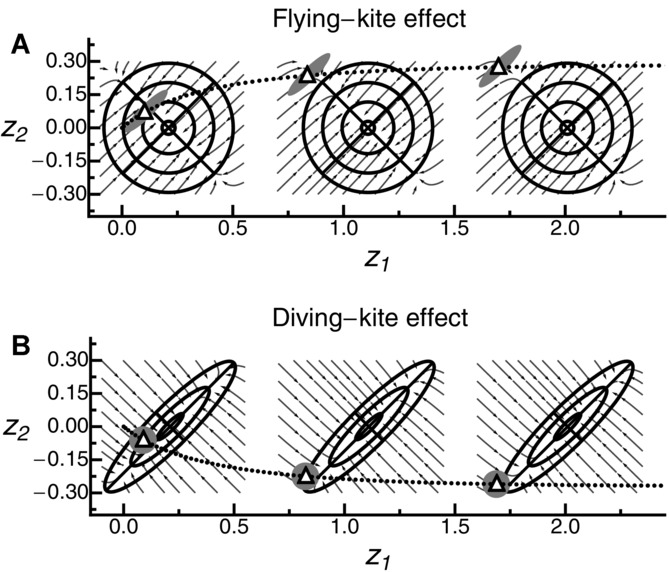
Schematic illustration of the flying-kite (top) and diving-kite effects (bottom) in a deterministic approximation, with snapshots taken at the initial (left), intermediate (middle), and equilibrium (right) phases. For each snapshot, the black ellipse represents the fitness landscape (defined by the Σ-matrix), the gray ellipse gives the shape of the distribution of new mutations (the M-matrix), the white triangle gives the current position of the mean phenotype, and dots show its trajectory. Gray arrows represent hypothetical trajectories toward a constant optimum if the population were placed at the beginning of the arrow. Results are based on the “canonical equation” of adaptive dynamics (Dieckmann and Law 1996), which states that the mean phenotype 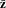 changes according to 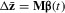, where 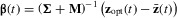 (Jones et al. 2004) is the selection gradient (which points in the direction of steepest ascent on the fitness landscape). (Note that the canonical equation is structurally identical to the Lande equation from quantitative genetics [Lande 1979, 1980; Jones et al. 2004] if the M-matrix is replaced by the G-matrix of standing genetic variation.) Without selectional correlation (top row), the selection gradient always points toward the current optimum. Without mutational correlation (bottom row), the selection gradient is parallel to the gray arrows.

Finally, mutational and selectional correlations also impact the trajectory of the mean phenotype (Figs.3, 5, S5_8; see also Jones et al. 2004). In particular, strong mutational correlations can cause the mean phenotype to trail above and behind the moving optimum—an effect that has been phrased the “flying-kite effect” (Jones et al. 2004). Conversely, with strong (positive) selectional correlation, the phenotypic mean follows the optimum behind and below. In analogy to the flying-kite effect, we call this phenomenon the “diving-kite effect.” Both effects can be explained by a deterministic model in which the change in the mean phenotype depends primarily on the leading eigenvector of **M** and the selection gradient β(*t*) (Fig. 7). Under strong mutational correlation, the change in mean phenotype is initially dominated by the leading eigenvector of **M**, causing the “rise of the kite,” until it is balanced by the selection gradient pointing toward the optimum. Under strong selectional correlation, however, the selection gradient is initially orthogonal to the leading eigenvector of Σ (i.e., the “ridge” of the fitness landscape), causing the mean phenotype to “dive.” Again, the trajectory will gradually change until it is aligned with the direction of the moving optimum (where it is aligned with the axis of largest width of the fitness landscape). Observing either the flying or the diving kite requires a sufficiently fast-changing environment (the kite needs to be pulled strongly enough) and at least intermediate levels of mutational or selectional correlations (right column Figs.5, S5_8). As the number of traits increases, the strength of both effects decreases on a per-trait basis, but their total strength increases (Supporting Information 4 and Fig. S4_1). Independently of the number of traits, the population on average takes smaller steps in the direction of the optimum as correlations (either selectional or mutational) become stronger (Figs. S5_2, S5_3).

### ACCURACY OF THE APPROXIMATIONS

Our main analytical tool has been the adaptive-walk approximation with normally distributed mutational effects. When compared to individual-based simulations of an explicit genetic model, its performance is often surprisingly good (e.g., Figs.4, 5, S5_2, S5_3, S5_8). Significant deviations occur mainly if the population-wide mutation rate Θ is high 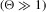, which, in violation of the adaptive-walk assumption, increases the probability of cosegregating beneficial mutations (Discussion and Figs. S5_9, S5_10). Because the adaptive-walk approximation does not account for population dynamics, it cannot be used to predict population persistence or extinction. Individual-based simulations show that long-term persistence is often impossible if the scaled rate of environmental change γ exceeds 0.1 (corresponding to *v*_1_ = 0.01 in Figs.6, S5_2, S5_3).

For slow environmental change, the normal distribution of new mutations can, furthermore, be approximated by an appropriate uniform distribution. The resulting approximation for adaptive walks works well for a broad range of small to intermediate rates of environmental change (see insets in Fig. S5_2). Naturally, this approximation cannot capture mutational correlations (see the poor fit for high values of ρ_*M*_ and *v*_1_ in Fig. S5_3). Note, however, that for sufficiently small rates of environmental change, mutational correlations can, indeed, be ignored (see above, Figs.4, 5).

Finally, we have attempted to approximate the distribution of adaptive substitutions over an entire adaptive walk by the distribution of the first step. This approximation works well in the one-trait case (Kopp and Hermisson 2009b), and in combination with a uniform distribution of new mutations, it is the only approach that allowed significant analytical progress (Supporting Information 4). With multiple traits, however, the first step makes a larger progress toward the optimum than the subsequent steps (Figs.2, S5_2, S5_3). The reason is that the first step will always introduce maladaptive pleiotropic side effects, which become compensated for by subsequent substitutions. Some of these compensatory substitutions are “backward steps,” which are beneficial, despite their effect being opposite to the direction of the optimum (e.g., Supporting Information 1, gray ellipse in Fig. S1_1 and “backward steps” in Figs. 1 and 4). Consequently, the first-step approximation works less well as the number of traits increases (Fig. S5_1). Furthermore, with mutational or selectional correlations, the direction of the first step systematically deviates from the distribution of step sizes over the entire adaptive walk (see flying- and diving-kite effects above; for the case of selectional correlation, see Fig. S5_11).

## Discussion

Environmental change forces populations to either adapt to the altered conditions or go extinct. In the absence of standing genetic variance for fitness, the outcome crucially depends on mutations, which provide the “genetic fuel” for adaptation, and selection that converts this resource into adaptive substitutions. Here, we have used analytical approximations and individual-based simulations to study the effects of pleiotropy or “organismic complexity” on the genetic basis of adaptation in gradually changing environments. In particular, we have investigated the distribution of adaptive substitutions (i.e., the distribution of the phenotypic effect sizes of fixed mutations) in populations following a moving optimum. Our results confirm and extend previous analysis of “adaptive walks” for single traits (Collins et al. 2007; Kopp and Hermisson 2007, 2009a,b). We show that the distribution of adaptive substitutions is largely determined by a single composite parameter γ, which scales the rate of environmental change relative to the “adaptive potential” of the population and defines a continuum between environmentally and genetically limited adaptation (Kopp and Hermisson 2009b). In the environmentally limited regime (slow environmental change), the population follows the optimum closely, adaptive steps are small and their multivariate distribution mirrors the shape of the fitness landscape. In the genetically limited regime, in contrast, the population follows the optimum with a large gap, adaptive steps are large and their distribution is determined primarily by the distribution of new mutations. We furthermore show that the mean effect size of fixed mutations increases with the degree of pleiotropy, in contrast to classical predictions from FGM under sudden environmental change. We now discuss these results in greater detail.

### THE EFFECT OF PHENOTYPIC COMPLEXITY ON THE GENETICS OF ADAPTATION

In complex organisms, pleiotropy is widespread—that is, most mutations affect multiple traits simultaneously. Different traits, therefore, do not evolve independently (Lande 1979; Agrawal and Stinchcombe 2009; Walsh and Blows 2009). With this basic fact in mind, Fisher (1930) used his classical geometric model to argue for a predominance of small mutations in adaptive evolution. Although theoretical studies later pointed out that large beneficial mutations may, nevertheless, play an important role (Kimura 1983; Gillespie 1993; Orr 1998, 2005a), they also confirmed that organisms pay a “cost of complexity” (Orr 1998, 2000; Welch and Waxman 2003) in the form of a reduced rate of adaptation. With regard to individual substitutions, Orr (2000) found that more complex organisms make smaller steps when adapting toward a fixed optimum (with step size measured as the decrease in the absolute distance to the optimum, which is closely related to the fitness effect of a fixed mutation). This is in direct contrast to our results for a moving optimum, where increased complexity leads to larger step sizes, with respect to both phenotype and fitness (Figs.2, S5_4).

The main reason for this finding arises from the ecological differences between the classical FGM and the moving-optimum model (consequences of different mutation models are discussed below). In the classical Fisher model, the proportion of beneficial mutations decreases with organismic complexity. Thus, the more phenotypic traits, the longer one has to wait for a beneficial mutation to appear (as adding another trait adds yet another dimension where mutations can go wrong; eq. S18). Of course, this argument still holds true under a moving optimum. As more complex organisms have to wait longer for a beneficial mutation to appear, the optimum has already traveled farther, enabling larger mutations to become fixed. Thus, the moving-optimum model does not contradict the “cost of complexity” argument, but reveals yet another aspect of it.

### ADAPTATION UNDER MUTATIONAL AND SELECTIONAL CORRELATIONS

The impact of mutational and selectional correlations on the distribution of adaptive substitutions is a direct consequence of the general principle that the shape of this distribution depends on the scaled rate of environmental change (see above). In particular, if the rate of environmental change is slow, only mutations from the very center of the mutational distribution can pass the selective sieve (Kopp and Hermisson 2009b), making mutational correlations irrelevant relative to the shape of the fitness landscape. Conversely, if adaptation is genetically limited, the selective sieve has less impact on the adaptive process than the supply with new mutations. Between these two extremes, the distribution of adaptive substitutions will progressively take the orientation of the mutational distribution as the rate of environmental change increases (Fig. 6).

Our results reveal strong parallels between the distribution of adaptive substitutions and the evolution of the **G**-matrix describing standing genetic variation (see also below). Recent quantitative-genetic studies have shown that both mutational and selectional correlations can induce correlations in the **G**-matrix, under both constant stabilizing and moving-optimum selection (Jones et al. 2003, 2004). A link between selectional correlation and genetic correlation has also been confirmed empirically (see Roff and Fairbairn 2012 for a recent meta-analysis). As shown in Figure S5_13, the distribution of adaptive substitutions closely matches the shape and orientation of the **G**-matrix. While this seems intuitive, it had not been shown by any previous study, and little is known about the relation between alleles in the standing variation and those that ultimately reach fixation (but see Hill 1982; Hill and Rasbash 1986a,b). This close correspondence between standing variation and fixed mutations might explain why the adaptive-walk approximation works surprisingly well even in populations with a high mutation rate (see Kopp and Hermisson 2009b). Quantitative-genetic studies have, so far, not systematically investigated how correlations in the **G**-matrix are affected by the rate of environmental change. It would be interesting to know whether the effects of mutational and selectional correlations on the **G**-matrix are similar to those on the distribution of adaptive substitutions.

Confirming previous results by Jones et al. (2004), our simulations showed that mutational and selectional correlations can cause systematic maladaption in traits under purely stabilizing selection (i.e., in directions orthogonal to the direction of the optimum). These “flying-” and “diving-kite” effects require that correlations are strong and the environmental change is sufficiently fast (i.e., in the genetically limited regime, see Figs.3 and 5). Strong effects are, therefore, likely to be restricted to a narrow parameter range, where populations might often be on the brink of extinction.

### DISCUSSION OF THE MODEL ASSUMPTIONS AND FUTURE DIRECTIONS

Like all models, our study is based on a number of simplifying assumptions, which might constrain the generality of our results. In the following, we discuss the likely consequences of these assumptions, potential extensions of the model, and ways to test our predictions empirically.

First, our model is based on the assumption of universal pleiotropy (Kacser and Burns 1981; for a review see Paaby and Rockman 2013). This assumption has been challenged recently, both because empirical levels of pleiotropy are rather low (median 1–7; Wang et al. 2010) and because true universal pleiotropy would induce unsustainably high costs (“the cost of complexity [...] should be more properly called the cost of pleiotropy”; Wagner and Zhang 2012, but see Hill and Zhang 2012a). Alternative approaches have, therefore, suggested modularity (Wagner and Altenberg 1996; Welch and Waxman 2003) or partial pleiotropy (Chevin et al. 2010; Lourenço et al. 2011) as a solution to this problem. Indeed, our model might be best interpreted as applying to a given module with a moderate level of pleiotropy. In any case, the relatively low number of traits assumed in most parts of this article is consistent with the degree of pleiotropy observed in natural populations (Martin and Lenormand 2006a,b; Wang et al. 2010). Thus, we expect our results to apply across a wide range of species facing environmental change.

Second, we assume the so-called Euclidean superposition model (Turelli 1985; Wagner 1988), where the distribution of mutational effects on a given trait is independent of complexity (see also Welch and Waxman 2003; Lourenço et al. 2011; Zhang 2012). Other studies (Orr 1998, 2000; Wingreen et al. 2003; and an alternative model in Welch and Waxman 2003) have instead used a “constant total-effects model,” in which the total mutational effect size (||α||) is constant across levels of complexity and, in consequence, the mean effect size on individual traits decreases. Indeed, this assumption explains part of our differences to Orr (1998, 2000). More generally, it raises the question of which factor is more important in shaping the distribution of adaptive substitutions at different levels of complexity: the “pleiotropic scaling” of mutations (Wagner et al. 2008) or the mode of environmental change. To address this issue, we conducted additional simulations, which combined constant and moving-optimum selection with the Euclidean superposition and constant total-effects models. These simulations yielded three main results (Fig. S5_12). First, the moving-optimum model behaves qualitatively similarly under both mutation models; in particular, average step size increases with complexity (at least as long as adaptation remains environmentally limited). This shows that our main results are robust to considerable variation in the pleiotropic scaling of mutational effects. Second, with a constant optimum, the mutation model does make a qualitative difference for the mean step size in the direction of the optimum (but not for total step size ||α||), which decreases with complexity under the constant total-effects model, but increases under the Euclidean superposition model. Third, a fundamental difference between constant and moving-optimum selection, which is independent of the mutation model, is seen at the level of the selection coefficients of fixed mutations, which decrease with complexity under a constant optimum but increase with complexity under a moving optimum. In summary, the mode of environmental change plays a fundamental role in shaping the distribution of adaptive substitutions and, in many cases, overrides the effects of pleiotropic scaling. Nevertheless, a better understanding of pleiotropic scaling—both empirically (Hermisson and McGregor 2008; Wagner et al. 2008; Wang et al. 2010; Wagner and Zhang 2011; Hill and Zhang 2012b) and with respects to its theoretical consequences—clearly is an important topic for future research.

Third, our adaptive-walk approximation assumes that evolution proceeds as a series of (hard) selective sweeps originating from new mutations. This follows the tradition of models based on a strong-selection–weak-mutation approximation (Gillespie 1983; Orr 1998, 2005a). In contrast, quantitative-genetic models assume that virtually all adaptation stems from standing genetic variation (in the context of the moving-optimum model, see, e.g., Bürger and Lynch 1995; Gomulkiewicz and Holt 1995; Jones et al. 2004, 2012; Zhang 2012; Chevin 2013; see also Fig. S5_13), and the importance of standing variation is well documented empirically (Hermisson and Pennings 2005; Barrett and Schluter 2008; Gomulkiewicz and Houle 2009; Teotónio et al. 2009; Jerome et al. 2011; Domingues et al. 2012; Messer and Petrov 2013). So far, quantitative-genetic models with a multidimensional moving optimum have focused either on the risk of population extinction (Gomulkiewicz and Houle 2009) or the maintenance and structure of genetic variation (Jones et al. 2004, 2012). To our knowledge, very little is known about the distribution of phenotypic effect sizes of adaptive substitutions when adaptation occurs from standing genetic variation. Because in this case the adaptive process is likely to have very different properties (e.g., adaptation could be faster with on average smaller mutations becoming fixed Barrett and Schluter 2008; Rockman 2012), this should be an important topic for future research.

Fourth, in accordance with the adaptive-walk approximation, most of our simulations (including those in Fig. 6) assumed a relatively (but not unrealistically) low population-wide mutation rate Θ. As such, we ignore effects of interactions between cosegregating beneficial mutations. For the one-dimensional case, Kopp and Hermisson (2009b) showed that high mutation rates in combination with low recombination (or a small number of loci) lead to an increase in the mean size of adaptive substitutions, due to Hill–Robertson interference (Hill and Robertson 1966; Gerrish and Lenski 1998). At high recombination rates (or with a large number of unlinked loci), in contrast, the mean step size decreases as a result of epistasis for fitness (due to stabilizing selection). Individual-based simulations suggest that these results also hold true for the multivariate case (Figs. S5_9, S5_10). Note, however, that the strength of interference is expected to decrease with increasing complexity, because the rate of beneficial mutations decreases.

In addition, Hill–Robertson interference also influences how the distribution of adaptive substitutions is affected by mutational and selection correlations. In particular, correlations between adaptive substitutions increase with increased linkage in the presence of mutational correlations (Fig. S5_9), but decrease with linkage in the presence of selectional correlations (Fig. S5_10). Thus, at high mutation rates, increasing linkage has a similar effect as increasing the scaled rate of environmental change γ. This makes intuitive sense, because interference weakens the efficiency of selection (Gerrish and Lenski 1998; Weissman and Barton 2012), which brings the adaptive process closer to the genetically limited regime.

Finally, our adaptive-walk approximation does not consider population dynamics. By setting an arbitrary extinction threshold with respect to mean fitness, we found that the maximal rate of environmental change a population can tolerate (Bürger and Lynch 1995), as well as the mean time to extinction, decreases with the number of traits (Fig. S5_5; this result is also supported by individual-based simulations). In particular, in complex organisms, long-term persistence in the face of an indefinitely moving optimum seems to be possible only in the environmentally limited regime (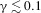), that is, when adaptation is not limited by the availability of new mutations. In the genetically limited regime, in contrast, populations can only persist for a limited amount of time (e.g., fast environmental change followed by a period of stasis). Over shorter time scales and with high mutation rates (large Θ, as in Jones et al. 2004), population persistence can also be facilitated by adaptation from standing genetic variation (Bürger and Lynch 1995; Barrett and Schluter 2008; Gomulkiewicz and Houle 2009). Indeed, our parameter γ is structurally similar to expressions describing the equilibrium phenotypic lag in quantitative-genetic models of adaptation to a moving optimum (eq. 5 in Jones et al. 2004; see also eq. 8a in Bürger and Lynch 1995). The interpretation is analogous: the lag increases with the speed of environmental change, and decreases with the strength of selection and the amount of (standing) genetic variation.

Our model makes a number of concrete predictions (Table 1) that can be tested empirically, even though such tests will certainly be challenging. The most direct approach is experimental evolution (for reviews see Elena and Lenski 2003; Kawecki et al. 2012; Barrick and Lenski 2013). Although the majority of studies have employed constant conditions (Reusch and Boyd 2013), Collins (2011b) recently urged for more studies in gradually changing environments. Microorganisms such as bacteria, yeast, or algae can be cultivated in media where an environmental factor such as temperature (Hietpas et al. 2013), salinity (Bell and Gonzalez 2009; Lachapelle and Bell 2012), pH (Hughes et al. 2007), the availability of nutrients (Collins 2011a), or the concentration of stressors such as antibiotics (Perron et al. 2008; Lindsey et al. 2013) or pollutants (Adamo et al. 2012) is gradually changed. Until now, these studies were mainly used to investigate the probability of “evolutionary rescue” (Gonzalez et al. 2013). Recent advances in sequencing technologies (reviewed in Metzker 2010), however, make it possible to conduct real-time genome-wide analyses and to map genetic changes to their effects on phenotype and fitness (Barrick et al. 2009; Barrick and Lenski 2013), such that the distribution of adaptive substitution over entire adaptive walks can be analyzed.

**Table 1:** A summary of theoretical predictions of the moving-optimum model

How does … affect adaptation?	Theoretical prediction
Mode of environmental change	
Sudden change	The distribution of adaptive substitutions is approximately exponential with respect to phenotype and fitness. Accordingly, most fixed mutations are of small effect and only a few large-effect alleles become fixed when approaching the constant optimum. The farther the optimum is away (i.e., the harsher the sudden environmental change) the larger the mutational effects that get fixed.
Gradual change	The distribution of adaptive substitutions with respect to phenotype (in the direction of the optimum or total effect) and fitness is gamma-like with an intermediate mode (Figs.1, S5_4). Thus, when following the moving optimum, most adaptive substitutions are of intermediate effect with only a few large-effect alleles becoming fixed.
Scaled rate of environmental change	The faster the rate of environmental change relative to the adaptive potential, the larger the mutational effects that become fixed. Holds true with respect to phenotype and fitness (Figs.2, S5_4). With increasing rate of environmental change the distribution of fitness effects becomes more asymmetric.
Complexity/Pleiotropy	Mean effect of adaptive substitutions with respect to phenotype and fitness increases as the number of traits affected by a single mutation increases (Figs.2, S5_12).
Mutational correlation	If the rate of environmental change is fast, the distribution of adaptive substitution mirrors the mutational distribution (Fig. 6).
Selectional correlation	If the rate of environmental change is slow, the distribution of adaptive substitution reflects the shape of the fitness landscape (Fig. 6).

In natural populations, where only present-day data are available, the most promising approach for studying the genetic basis of adaptation is the analysis of quantitative-trait loci (QTLs) in diverging populations. For example, Langlade et al. (2005) identified QTLs for leaf shape in two species of *Antirrhinum* and postulated a sequence of substitutions that can traverse the “allometric space” between them. Albert et al. (2008) and Rogers et al. (2012) analyzed genetic differences between ancestral marine and derived freshwater populations of sticklebacks (*Gasterosteus aculeatus*). Rogers et al. (2012) compared two sets of freshwater populations and showed that those whose environment (and presumably phenotypic optimum) is more different from that of the marine populations (with respect to salinity and presence of predators) displayed a higher frequency of large-effect QTLs. Albert et al. (2008) crossed an ancestral Pacific and a highly derived benthic freshwater form and found a gamma-like distribution of QTL effect sizes with an intermediate mode. Taking into account the detection limits for small-effect QTLs (Otto and Jones 2000), they interpreted this result as support for FGM with constant selection (i.e., the difficulty in identifying small QTLs would turn the predicted exponential distribution into an observed gamma-like distribution). However, both studies could, in principle, also be interpreted as showing the outcome of adaptation to a moving optimum (as briefly discussed in Schluter et al. 2010; Rogers et al. 2012), which directly predicts a distribution of effect sizes with an intermediate mode. More stringent tests of the present theory would require studying populations for which a moving optimum can be assumed a priori (e.g., comparisons of microalgae from pristine habitats with populations known to have experienced gradual eutrophication). Even then, the difficulty in detecting small-effect substitutions will remain a major challenge (Otto and Jones 2000).

### CONCLUSION

Natural populations are constantly forced to adapt to changing environments, a process that takes place in a high-dimensional phenotype and genotype space. Along with previous studies, our analysis of the moving-optimum model shows that the genetic basis of this process depends critically on the tempo and mode of environmental change. In particular, our environmentally and genetically limited regimes lead to qualitative differences in the distribution of adaptive substitution, with respect to its mean, shape, and correlation patterns. Long-term persistence is likely restricted to the environmentally limited regime—where adaptation proceeds “smoothly” in small steps—but the parameter range for this regime is reduced in complex organisms.

## Supporting Information

Supporting Information 1 . The selection coefficient. **Figure S1_1**. The time dependence of selection in the two-dimensional moving-optimum model. **Supporting Information 2**. Transformation of phenotype space. **Supporting Information 3**. The parameter γ. **Supporting Information 4**. The environmentally-limited regime. **Figure S4_1**. Dependence of various components of the first adaptive step  in the transformed phenotype space, as a function of the scaled rate of environmental change γ for various numbers of traits *n*, assuming a uniform distribution of new mutations. **Figure S4_2**. Coefficient of variation of the size of the first step in the direction of the moving optimum, . **Supporting Information 5**. Supplementary figures. **Figure S5_1**. Distribution of the size of the first adaptive substitution for two (top row) and four (bottom row) traits and two different rates of environmental change *v*_1_. **Figure S5_2**. Mean size  of adaptive substitutions in the direction of the moving optimum for the first step (lines) and over the entire adaptive walk (symbols), as a function of the rate of environmental change *v*_1_, for *n* = 2 and three traits and different values of selectional correlation ρ_*M*_. **Figure S5_3**. Mean size  of adaptive substitutions in the direction of the moving optimum, for the first step (lines) and over the entire adaptive walk (symbols), as a function of the rate of environmental change *v*_1_, for *n* = 2 and three traits and different values of mutational correlation ρ_*M*_. **Figure S5_4**. Mean selection coefficient of adaptive substitutions as a function of the rate of environmental change for various strengths of mutational (dashed lines) and selectional (solid lines) correlations ρ_*M*_ and ρ_Σ_. **Figure S5_5**. The mean time to extinction for different levels of phenotypic complexity *n* as a function of the rate of environmental change. **Figure S5_6**. The multivariate distribution of the first adaptive substitution (left) and over the entire adaptive walk (right) for *n* = 2 traits, when the optimum moves slowly in the direction of the first trait and the effects of new mutations are strongly correlated (ρ_*M*_ = 0.9). **Figure S5_7**. The multivariate distribution of the first adaptive substitution (left) and over the entire adaptive walk (right) for *n* = 2 traits, when the optimum moves slowly in the direction of the first trait and selection is strongly correlated (ρ_Σ_ = 0.9). **Figure S5_8**. The impact of mutational and selectional correlations on the distribution of adaptive substitutions for *n* = 3 traits. For details, see Figure of the main text. **Figure S5_9**. The effects of linkage and interference between cosegregating alleles on the mean step size in direction of the moving optimum α_1_ (left) and the correlation between adaptive substitutions (right) under strong mutational correlations ρ_*M*_ = 0.9. **Figure S5_10**. The effects of linkage and interference between cosegregating alleles on the mean step size in direction of the moving optimum α_1_ (left) and the correlation between adaptive substitutions  (right) under strong selectional correlations ρ_Σ_ = 0.9. **Figure S5_11**. The multivariate distribution of the first adaptive substitution in adaptive-walk simulations with strong selectional correlation (ρ_Σ_ = 0.9), illustrating the diving-kite effect (the negative bias in the α_2_-direction) present for fast (*v*_1_ = 0.1) but not for slow (*v*_1_ = 10^−5^) environmental change. **Figure S5_12**. Comparison of the first adaptive substitution under the constant total effect (darker gray) and the Euclidean superposition model (lighter gray) for Fisher's geometric model with constant selection (left) and a moving optimum (right). **Figure S5_13**. The **G**-matrix (gray ellipse) and the 90% confidence ellipse of the distribution of adaptive substitutions (dark ellipse) under strong mutational (ρ_*M*_ = 0.9) and selectional (ρ_Σ_ = 0.9) correlation for various rates of environmental change (*v*_1_).
