# Supplementary material for: FISHER'S GEOMETRIC MODEL WITH A MOVING OPTIMUM
Source: Evolution. 2014 Jul 10;68(9):2571–88. doi: 10.1111/evo.12465 (PMC4285815; doi:10.1111/evo.12465)
Supplement: Supporting Information 1 — . The selection coefficient. Figure S1_1. The time dependence of selection in the two-dimensional moving-optimum model. Supporting Information 2. Transformation of phenotype space. Supporting Information 3. The parameter γ. Supporting Information 4. The environmentally-limited regime. Figure S4_1. Dependence of various components of the first adaptive step in the transformed phenotype space, as a function of the scaled rate of environmental change γ for various numbers of traits n, assuming a uniform distribution of new mutations. Figure S4_2. Coefficient of variation of the size of the first step in the direction of the moving optimum, . Supporting Information 5. Supplementary figures. Figure S5_1. Distribution of the size of the first adaptive substitution for two (top row) and four (bottom row) traits and two different rates of environmental change v1. Figure S5_2. Mean size of adaptive substitutions in the direction of the moving optimum for the first step (lines) and over the entire adaptive walk (symbols), as a function of the rate of environmental change v1, for n = 2 and three traits and different values of selectional correlation ρM. Figure S5_3. Mean size of adaptive substitutions in the direction of the moving optimum, for the first step (lines) and over the entire adaptive walk (symbols), as a function of the rate of environmental change v1, for n = 2 and three traits and different values of mutational correlation ρM. Figure S5_4. Mean selection coefficient of adaptive substitutions as a function of the rate of environmental change for various strengths of mutational (dashed lines) and selectional (solid lines) correlations ρM and ρΣ. Figure S5_5. The mean time to extinction for different levels of phenotypic complexity n as a function of the rate of environmental change. Figure S5_6. The multivariate distribution of the first adaptive substitution (left) and over the entire adaptive walk (right) for n = 2 traits, when the optimum moves slowly in the dir [file evo0068-2571-sd1.pdf]

Supporting information to "Fisher's geometric model with a moving optimum"

Sebastian Matuszewski Joachim Hermisson
Michael Kopp

Supporting Information 1: The selection coefficient

Figure **S1** illustrates the time dependence of the selection coefficient $s(\mathbf{x}, \mathbf{y}, t)$ in the multi-dimensional moving-optimum model. Recall that $s(\mathbf{x}, \mathbf{y}, t)$ can be written as

$$s\left(\mathbf{x}, \mathbf{y}, t\right) \approx \lambda_{\mathbf{x},\mathbf{y}} \left(t - \tau_{\mathbf{x},\mathbf{y}}\right), \tag{S1a}$$

with

$$\lambda_{\mathbf{x},\mathbf{y}} = 2(\mathbf{x} - \mathbf{y})'\mathbf{\Sigma}^{-1}\mathbf{v} \tag{S1b}$$

$$\tau_{\mathbf{x},\mathbf{y}} = \frac{(\mathbf{x} - \mathbf{y})'\mathbf{\Sigma}^{-1}(\mathbf{x} + \mathbf{y})}{2(\mathbf{x} - \mathbf{y})'\mathbf{\Sigma}^{-1}\mathbf{v}}, \tag{S1c}$$

(provided $\lambda_{\mathbf{x},\mathbf{y}} \neq 0$), where $\lambda_{\mathbf{x},\mathbf{y}}$ is the rate of change and $\tau_{\mathbf{x},\mathbf{y}}$ is the lag time (i.e., the time when s reaches zero).

The line $\lambda_{\mathbf{x},\mathbf{y}} = 0$ contains all mutational effects $\boldsymbol{\alpha}$ that are orthogonal to $\mathbf{\Sigma}^{-1}\mathbf{v}$, which in the case of uncorrelated selection ($\rho_\Sigma = 0$) simply means orthogonal to the direction of the moving optimum. Accordingly, the line divides the space of mutant phenotypes into "backward mutations" ($\lambda_{\mathbf{x},\mathbf{y}} < 0$), which have a chance at fixation only during a limited time window (if any), and "forward mutations", which have an unlimited amount of time to appear and go on to fixation. The set of mutations

that are beneficial at time t is given by an ellipse (the solution of $s(\mathbf{x}, \mathbf{y}, t) = 0$) that passes through the wild-type $\mathbf{y}$ and has its center at the current optimum $\mathbf{v}t$. Note that, as the optimum moves on, the area of this ellipse decreases as long as the optimum is to the left of the $\lambda_{\mathbf{x},\mathbf{y}} = 0$ line, and increases indefinitely afterwards. Together with the $\lambda_{\mathbf{x},\mathbf{y}} = 0$ line, the two ellipses corresponding to the initial and the current optimum (bold and gray ellipse in Fig. **S1**, respectively) split the space of mutant phenotypes into six sectors: backward mutations that never were and never will be beneficial (sector **I**); backward mutations that were beneficial initially, but which have become deleterious by that time (sector **II**); backward mutations that are still beneficial (sector **III**); forward mutations that have been beneficial from the outset (sector **IV**); forward mutations that have become beneficial after a positive lag time $\tau_{\mathbf{x},\mathbf{y}}$ (sector **V**); and forward mutations that are not yet beneficial but will become beneficial in the future (sector **VI**). Note that, as the optimum moves on, sectors **II** and **V** will grow, sectors **III** and **VI** will shrink, and sectors **I** and **IV** remain unchanged.

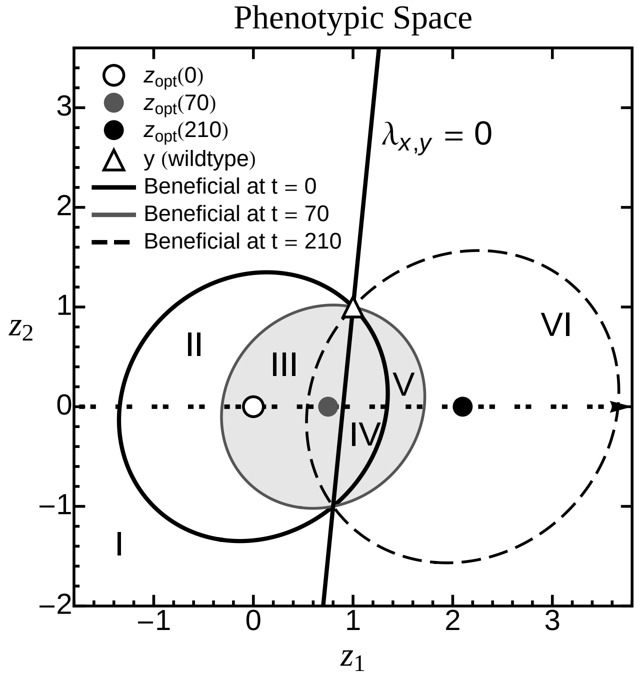

Figure S1: The time dependence of selection in the two-dimensional moving optimum model. The axes span the values of two quantitative traits. The wildtype phenotype combination $\mathbf{y}$ is represented by the open triangle, and the optimum $\mathbf{z}_{\text{opt}}$ has moved at constant speed along the dotted line from the open circle at time $t = 0$ to the grey circle at time $t = 70$ and the black circle at time $t = 210$. The solid ellipse encloses the set of mutant phenotypes that were selectively favored at $t = 0$ (i.e., $\{\mathbf{x} \mid s(\mathbf{x}, \mathbf{y}, 0) > 0\}$), whereas the grey and the dashed ellipses represent those mutants that are selectively favored at $t = 70$ and $t = 210$, respectively ($\{\mathbf{x} \mid s(\mathbf{x}, \mathbf{y}, 70) > 0\}$). The solid line is the line $\lambda_{\mathbf{x},\mathbf{y}} = 0$, which divides the phenotype space into "forward" and "backward" types as described in the text. The roman numerals refer to sets of mutant phenotypes at time $t = 70$ that differ with respect to their past, present and future selection coefficient (see text for details). Parameters: $v_1 = 0.01, \Theta = 2, \sigma^2 = 10, \rho_\Sigma = 0.1$.

Supporting Information 2: Transformation of phenotype space

To make further progress, we introduce a transformation of the phenotype space, which will be denoted by tildes ($\tilde{\mathbf{z}}$ etc.) and has the following properties: (i) the selection matrix $\tilde{\mathbf{\Sigma}}$ is proportional to the identity matrix, that is, selection is equally strong in all directions (isotropic); (ii) the optimum moves in the direction of the first trait axis, $\tilde{\mathbf{v}} = (\tilde{v}_1, 0, \ldots)'$, that is, only the first trait is under moving-optimum selection, whereas all other traits are under constant stabilizing selection; (iii) the mutation matrix $\tilde{\mathbf{M}}$ has determinant 1; if mutation is uncorrelated in the transformed space, this means that the geometric mean of the mutational standard deviations equals 1; therefore, the length scale is determined by the average size of new mutations. According to these goals, the transformation is done in three steps. First, let $\mathbf{A}$ be the matrix whose rows contain the eigenvectors of $\mathbf{\Sigma}$, scaled to magnitude 1, and let $\mathbf{D}$ be the diagonal matrix containing the corresponding eigenvalues (i.e., $\mathbf{\Sigma}^{-1} = \mathbf{A}'\mathbf{D}^{-1}\mathbf{A}$). For the first step of the transformation, we define

$$\mathbf{B} = \bar{\sigma}\mathbf{D}^{-1/2}\mathbf{A}, \tag{S2}$$

with $\bar{\sigma} = \sqrt[2n]{\det(\mathbf{\Sigma})}$ (eq. 3), such that $\det(\mathbf{B}) = 1$ and $\mathbf{B}'\mathbf{B} = \bar{\sigma}^2\mathbf{\Sigma}^{-1}$. Substituting $\mathbf{z}$ by $\mathbf{B}^{-1}\dot{\mathbf{z}}$, such that $\dot{\mathbf{z}} = \mathbf{B}\mathbf{z}$, $\dot{\mathbf{x}} = \mathbf{B}\mathbf{x}$, $\dot{\mathbf{y}} = \mathbf{B}\mathbf{y}$, $\dot{\mathbf{v}} = \mathbf{B}\mathbf{v}$ and using the fact that $\tilde{\mathbf{\Sigma}}^{-1} = (\mathbf{B}^{-1})'\mathbf{\Sigma}^{-1}\mathbf{B}^{-1} = \bar{\sigma}^{-2}\mathbf{I}$, the selection coefficient (eq. 8a) in the transformed phenotype space is given by

$$\dot{s}(\dot{\mathbf{x}}, \dot{\mathbf{y}}, t) = \bar{\sigma}^{-2}\left(\|\dot{\mathbf{y}} - \dot{\mathbf{v}}t\|^2 - \|\dot{\mathbf{x}} - \dot{\mathbf{v}}t\|^2\right) \tag{S3}$$

where $\|\xi\|^2 = \xi'\xi$ is the square of the Euclidean norm. After this first transformation step, goal (i) has been reached, that is, selection is symmetric in all directions with strength $\bar{\sigma}^{-2}$. For the second step, we need to define a $n \times n$ rotation matrix $\mathbf{R}$ that satisfies $\mathbf{R}\dot{\mathbf{v}} = (\|\dot{\mathbf{v}}\|, 0, \ldots, 0)'$. For the present calculation, it is not necessary to give $\mathbf{R}$ explicitly. However, for numerical calculations, such a matrix can always been found by applying the Gram-Schmidt orthonormalization algorithm (and a potential step of rearrangement) to a basis of the unrotated vector space that is given by the $n \times n$ identity matrix whose i^{th} column is replaced by $\dot{\mathbf{v}}$, where i is determined by the first non-zero entry of $\dot{\mathbf{v}}$. Like all rotation matrices, $\mathbf{R}$ satisfies $\mathbf{R}' = \mathbf{R}^{-1}$ and $\det(\mathbf{R}) = 1$. With the transformations $\ddot{\mathbf{z}} = \mathbf{R}\dot{\mathbf{z}}$ etc., we get

$$\ddot{s}(\ddot{\mathbf{x}}, \ddot{\mathbf{y}}, t) = \bar{\sigma}^{-2}\left(\|\ddot{\mathbf{y}} - \ddot{\mathbf{v}}t\|^2 - \|\ddot{\mathbf{x}} - \ddot{\mathbf{v}}t\|^2\right). \tag{S4}$$

The third step of the transformation is to express all vectors relative to $\bar{m} = \sqrt[2n]{\det(\mathbf{M}^{-1})}$ (eq. 5), that is, $\tilde{\mathbf{z}} = \ddot{\mathbf{z}}/\bar{m}$ etc., leading to

$$\tilde{s}(\tilde{\mathbf{x}}, \tilde{\mathbf{y}}, t) = \bar{\tilde{\sigma}}^{-2} \left(\|\tilde{\mathbf{y}} - \tilde{\mathbf{v}}t\|^2 - \|\tilde{\mathbf{x}} - \tilde{\mathbf{v}}t\|^2 \right). \tag{S5}$$

with $\bar{\tilde{\sigma}} = \bar{\sigma}/\bar{m}$. Summarizing, we can combine all three steps by defining a transformation matrix

$$\mathbf{Q} = \frac{1}{\bar{m}}\mathbf{RB} = \bar{\tilde{\sigma}}\mathbf{RD}^{-1/2}\mathbf{A}, \tag{S6}$$

with $\det(\mathbf{Q}) = \bar{m}^{-n} = \sqrt{\det(\mathbf{M}^{-1})}$, such that $\tilde{\mathbf{z}} = \mathbf{Qz}$ etc. Note that the transformation also affects the distribution of new mutations $\tilde{p}(\tilde{\boldsymbol{\alpha}})$, which is given by $\tilde{p}(\tilde{\boldsymbol{\alpha}}) = \det(\mathbf{Q}^{-1}) \cdot p(\mathbf{Q}^{-1}\tilde{\boldsymbol{\alpha}}) = \bar{m}^n p(\boldsymbol{\alpha})$, and has covariance matrix $\tilde{\mathbf{M}} = \mathbf{QMQ}'$ with $\det(\tilde{\mathbf{M}}) = 1$.

Supporting Information 3: The parameter γ

We can use the transformation from Supporting Information 2 to show that the parameters Θ, $\mathbf{v}$ and $\boldsymbol{\Sigma}$ affect the distribution of adaptive substitutions only through the composite parameter γ.

First, using the fact that $\tilde{\mathbf{v}} = (\tilde{v}_1, 0, \ldots)'$, $g(t, \mathbf{y})$ (eq. 9) can be rewritten as

$$\tilde{g}(t, \tilde{\mathbf{y}}) = \Theta\bar{\tilde{\sigma}}^{-2} \int_{\tilde{\chi}} \tilde{p}(\tilde{\boldsymbol{\alpha}}) \left[2(\tilde{x}_1 - \tilde{y}_1)'\tilde{v}_1 t - (\tilde{\mathbf{x}}'\tilde{\mathbf{x}} - \tilde{\mathbf{y}}'\tilde{\mathbf{y}}) \right] d\tilde{\mathbf{x}}, \tag{S7}$$

where the integration region $\tilde{\chi}(t, \tilde{\mathbf{y}}) = \{\tilde{\mathbf{x}} \mid \|\tilde{\mathbf{x}} - \tilde{\mathbf{v}}t\| < \|\tilde{\mathbf{y}} - \tilde{\mathbf{v}}t\|\}$ is the set of mutant phenotypes in the transformed space with positive selection coefficient at time t. Next, using the substitution $\zeta = \tilde{v}_1 t$, the waiting-time distribution on the transformed scale, $\tilde{F}(t|\tilde{\mathbf{y}}) = \exp\left(-\int_0^t \tilde{g}(\tau, \tilde{\mathbf{y}}) d\tau\right)$ (eq. 10) can be written as

$$\tilde{F}(t|\tilde{\mathbf{y}}) = \exp\left(-\frac{1}{\gamma} \int_0^{\tilde{v}_1 t} \int_{\tilde{\chi}} \tilde{p}(\tilde{\boldsymbol{\alpha}}) \left[2(\tilde{x}_1 - \tilde{y}_1)'\zeta - (\tilde{\mathbf{x}}'\tilde{\mathbf{x}} - \tilde{\mathbf{y}}'\tilde{\mathbf{y}}) \right] d\tilde{\mathbf{x}} d\zeta \right) \tag{S8}$$

with

$$\gamma = \frac{\tilde{v}_1}{\Theta\tilde{\sigma}^{-2}}. \tag{S9}$$

Therefore, $\tilde{F}(t|\tilde{\mathbf{y}})$ depends only on γ, $\tilde{p}$ (or $\tilde{\mathbf{M}}$), $\tilde{\mathbf{y}}$, and the product $\tilde{v}_1 t$. Furthermore, the same substitution can be applied to the distribution of adaptive substitutions,

which can be written as

$$\tilde{\phi}(\tilde{\boldsymbol{\alpha}}|\tilde{\mathbf{y}}) = \frac{\tilde{p}(\tilde{\boldsymbol{\alpha}})}{\gamma} \int_{\frac{\tilde{\mathbf{x}}'\tilde{\mathbf{x}}-\tilde{\mathbf{y}}'\tilde{\mathbf{y}}}{2(\tilde{x}_1-\tilde{y}_1)}}^{\infty} \left[2(\tilde{x}_1 - \tilde{y}_1)\zeta - (\tilde{\mathbf{x}}'\tilde{\mathbf{x}} - \tilde{\mathbf{y}}'\tilde{\mathbf{y}})\right] \tilde{F}(\zeta|\tilde{\mathbf{y}}) d\zeta. \tag{S10}$$

Thus, in the transformed space, the distribution of adaptive substitutions depends only on γ, the initial phenotype $\tilde{\mathbf{y}}$, and the distribution of new mutations $\tilde{p}(\tilde{\boldsymbol{\alpha}})$. At the original scale, we have $\phi(\boldsymbol{\alpha}|\mathbf{y}) = \det(\mathbf{Q})\tilde{\phi}(\tilde{\boldsymbol{\alpha}}|\tilde{\mathbf{y}}) = \bar{m}^{-n}\tilde{\phi}(\mathbf{Q}\boldsymbol{\alpha}|\mathbf{Q}\mathbf{y})$. Finally, since

$$\tilde{v}_1 = \|\tilde{\mathbf{v}}\| = \frac{1}{\bar{m}}\|\mathbf{B}\mathbf{v}\| = \frac{1}{\bar{m}}\sqrt{\mathbf{v}'\mathbf{B}'\mathbf{B}\mathbf{v}} = \bar{\bar{\sigma}}\sqrt{\mathbf{v}'\boldsymbol{\Sigma}^{-1}\mathbf{v}}, \tag{S11}$$

γ reduces to the form given in equation (13) of the main text when expressed in terms of the original variables.

In the adaptive-walk approximation, the effects of the rate and direction of environmental change $\mathbf{v}$, the population-wide mutation rate Θ and the selection matrix $\boldsymbol{\Sigma}$ are completely captured by γ. The same is, however, not true for changes in the mutational covariance matrix $\mathbf{M}$ (and, hence, the distribution of new mutations $p(\boldsymbol{\alpha})$; see eq. S10), since γ contains only the "average variance" of mutational effects ($\bar{m}^2$, eq. 5), but not the details of the correlation structure. γ also does not capture the impact of organismic complexity *per se*, as it is independent of n in the absence of mutational and selectional correlations (eq. 14). In the presence of correlations, γ may depend on n, but only because $\bar{\sigma}$ or $\bar{m}$ depend on n. For example, increasing the number of selectionally correlated traits increases the average strength of selection, and hence decreases $\bar{\sigma}$ (eq. 3).

Kopp and Hermisson (2009) proposed a value of $\gamma \ll 1$ as an approximate boundary between the environmentally- and genetically-limited regimes. In the context of the present paper, a value of $\gamma = 1$ is already very large and often leads to population extinction in individual-based simulations. Indeed, it refers to a situation where the adaptive process is clearly neither environmentally nor genetically limited. For $n = 1$, the environmental limit (where the distribution of new mutations can be treated as effectively uniform) provides a very good approximation for $\gamma \lesssim 10^{-2} - 10^{-1}$ (see Fig. 4F Kopp and Hermisson 2009). Here, we show that this boundary shifts to smaller values as complexity increases (reflecting the cost of complexity, see above), but the approximation remains reasonably good for $\gamma = 10^{-2}$ (Fig. S**5**_1, S**5**_2, S**5**_3). In general, for the mean step size in the direction of the optimum ($\bar{\alpha}_1$), the relative error incurred by the approximation remains at the order of 10% as long as the mean step size is on the order of magnitude of the (mean) standard deviation

of the effects of new mutations ($\bar{\alpha}_1 \approx \bar{m}$, see Fig S**5**_2). Similarly, in Fig. 6, the effects of mutational and selectional correlations offset each other (indicating an intermediate regime) around $\gamma = 0.1$ ($v_1 \approx 0.005$).

Supporting Information 4: The environmentallylimited regime (uniform distribution of new mutations)

As argued in the main text, if γ is sufficiently small, the distribution of new mutations $p(\boldsymbol{\alpha})$ can be approximated by a uniform distribution with $p_u(\boldsymbol{\alpha}) = p(\mathbf{0}) \equiv p_0$. This simplification allows further analytical progress. For the instantaneous rate of fixation, $\tilde{g}(t, \tilde{\mathbf{y}})$ (eq. S7), we can use the fact that, in the transformed phenotype space, all mutants with a given distance $\tilde{r} = \|\tilde{\mathbf{x}} - \tilde{\mathbf{v}}t\|$ from the optimum have identical selection coefficients

$$\tilde{s}(\tilde{r}, \tilde{\mathbf{y}}, t) = \bar{\tilde{\sigma}}^{-2}\left(\tilde{\delta}(t, \tilde{\mathbf{y}})^2 - \tilde{r}^2\right), \quad \text{for } \tilde{r} < \tilde{\delta}(t, \tilde{\mathbf{y}}) \tag{S12}$$

where we denote by $\tilde{\delta}(t, \tilde{\mathbf{y}}) = \|\tilde{\mathbf{y}} - \tilde{\mathbf{v}}t\|$ the distance of the wild-type from the optimum at time t.

The weight of such a class of mutants is given by the surface $S_n(\tilde{r})$ of a n-dimensional hypersphere with radius $\tilde{r}$ (Hartl and Taubes 1998; Tenaillon et al. 2007; Gros et al. 2009), which is

$$S_n = \frac{2\pi^{\frac{n}{2}}}{\Gamma\left(\frac{n}{2}\right)}\tilde{r}^{n-1}, \tag{S13}$$

where $\Gamma(\bullet)$ denotes the gamma function. $\tilde{g}(t, \tilde{\mathbf{y}})$ is then given by

$$\begin{aligned}
\tilde{g}(t, \tilde{\mathbf{y}}) &= \Theta\tilde{p}_0 \int_0^{\tilde{\delta}(t,\tilde{\mathbf{y}})} S_n(\tilde{r}) s(\tilde{r}, \tilde{\mathbf{y}}, t) d\tilde{r} \\
&= \Theta\tilde{p}_0\bar{\tilde{\sigma}}^{-2}\frac{2\pi^{\frac{n}{2}}}{\Gamma\left(\frac{n}{2}\right)} \int_0^{\tilde{\delta}(t,\tilde{\mathbf{y}})} \tilde{r}^{n-1}\left(\tilde{\delta}(t, \tilde{\mathbf{y}})^2 - \tilde{r}^2\right) d\tilde{r} \\
&= \Theta\tilde{p}_0\bar{\tilde{\sigma}}^{-2}\frac{\pi^{\frac{n}{2}}}{\Gamma\left(2 + \frac{n}{2}\right)}\tilde{\delta}(t, \tilde{\mathbf{y}})^{n+2},
\end{aligned} \tag{S14}$$

where we used the fact that $\xi\Gamma(\xi) = \Gamma(\xi + 1)$. Here, $\tilde{p}_0 = \det(\mathbf{Q}^{-1})p_0 = \bar{m}^n p_0$. For

a general wild-type phenotype $\mathbf{y}$, the waiting-time distribution is given by

$$\tilde{F}(t|\tilde{\mathbf{y}}) = \exp\left(-\int_0^t \tilde{g}(\tau, \tilde{\mathbf{y}})d\tau\right) = \exp\left(-\frac{\tilde{p}_0}{\gamma}\frac{\pi^{\frac{n}{2}}}{\Gamma\left(2+\frac{n}{2}\right)}\tilde{v}_1\int_0^t \|\tilde{v}_1\tau - \tilde{\mathbf{y}}\|^{n+2}d\tau\right). \tag{S15}$$

The integral in the exponent can be easily calculated for even values of n, and (e.g., by using *Mathematica*) also for small odd n, but the resulting expressions are unwieldy and will not be given here. We note that adaptive walks in the environmentally-limited regime can be efficiently simulated by alternately drawing the step time from the distribution (S15) and the step size from the conditional distribution (11). (For the latter, use the symmetry of the transformed trait space by first drawing the new distance from the optimum, $\tilde{r}$ [see (S14)], and then choosing a random direction.)

Characterization of the first step of the adaptive walk in the environmentally-limited case. Further progress can be made if the wild-type is initially well-adapted ($\mathbf{y} = \mathbf{0}$), such that $\tilde{\delta}(t, \tilde{\mathbf{y}}) = \tilde{v}_1 t$. We refer to the adaptive substitution with these initial conditions as the first step of the adaptive walk. The waiting-time distribution for this step simplifies to

$$\tilde{F}(t|\mathbf{0}) = \exp\left(-\frac{\tilde{p}_0}{\gamma}\eta(n)(\tilde{v}_1 t)^{n+3}\right) \tag{S16}$$

with

$$\eta(n) = \frac{\pi^{\frac{n}{2}}}{(n+3)\Gamma\left(2+\frac{n}{2}\right)}. \tag{S17}$$

Below, we will use the moments of the waiting-time distribution, which are given by

$$\mathrm{E}(t^i|\mathbf{0}) = \int_0^\infty t^i \tilde{f}(t|\mathbf{0})dt = \frac{1}{\tilde{v}_1^i}\left(\frac{\gamma}{\eta(n)\tilde{p}_0}\right)^{\frac{i}{n+3}}\Gamma\left(\frac{n+3+i}{n+3}\right), \quad i = 1, 2, \dots. \tag{S18}$$

Using equations (S1) and (12b), the distribution of the first adaptive step can be

expressed as

$$\tilde{\phi}(\tilde{\boldsymbol{\alpha}}|\mathbf{0}) = \lambda_{\tilde{\boldsymbol{\alpha}},\mathbf{0}}\tau^2_{\tilde{\boldsymbol{\alpha}},\mathbf{0}}\left(\mathbf{E}_{\frac{1+n}{3+n}}\left(\frac{\eta(n)\tilde{p}_0}{\gamma}\left(\tilde{v}_1\tau_{\tilde{\boldsymbol{\alpha}},\mathbf{0}}\right)^{n+3}\right) - \mathbf{E}_{\frac{2+n}{3+n}}\left(\frac{\eta(n)\tilde{p}_0}{\gamma}\left(\tilde{v}_1\tau_{\tilde{\boldsymbol{\alpha}},\mathbf{0}}\right)^{n+3}\right)\right)$$

$$= \frac{\tilde{p}_0}{(n+3)\gamma}\frac{\|\tilde{\boldsymbol{\alpha}}\|^4}{2\tilde{\alpha}_1}\left(\mathbf{E}_{\frac{1+n}{3+n}}\left(\frac{\eta(n)\tilde{p}_0}{\gamma}\left[\frac{\|\tilde{\boldsymbol{\alpha}}\|^2}{2\tilde{\alpha}_1}\right]^{n+3}\right) - \mathbf{E}_{\frac{2+n}{3+n}}\left(\frac{\eta(n)\tilde{p}_0}{\gamma}\left[\frac{\|\tilde{\boldsymbol{\alpha}}\|^2}{2\tilde{\alpha}_1}\right]^{n+3}\right)\right), \tag{S19}$$

where $\mathbf{E}_\xi(\psi) = \int_1^\infty (\exp(-\psi t)/t^\xi)dt$ denotes the exponential integral function.

While this expression is not particularly instructive, further insight can be gained by focusing on the moments of the distributions of certain components of $\tilde{\boldsymbol{\alpha}}$.

Distribution of $\tilde{\alpha}_1$. We start by deriving the moments of the marginal distribution of step sizes in the direction of the optimum, which we will denote by $\tilde{\phi}_1(\tilde{\alpha}_1)$. Here and below, our strategy will be to first focus on the conditional distribution of step sizes given the waiting time (eq. 11), which in the transformed trait space, is given by

$$\tilde{\phi}(\tilde{\boldsymbol{\alpha}}|t,\mathbf{0}) = \frac{\Theta\tilde{p}_0 s(\tilde{\boldsymbol{\alpha}},\mathbf{0},t)}{\tilde{g}(t,\mathbf{0})} = \frac{2\tilde{\alpha}_1\tilde{v}_1 t - \|\tilde{\boldsymbol{\alpha}}\|^2}{\frac{\pi^{n/2}}{\Gamma\left(2+\frac{n}{2}\right)}(\tilde{v}_1 t)^{n+2}}. \tag{S20}$$

Accordingly, the conditional distribution of $\tilde{\alpha}_1$ is

$$\begin{aligned}\tilde{\phi}_1(\tilde{\alpha}_1|t,\mathbf{0}) &= \int_0^{\sqrt{2\tilde{\alpha}_1\tilde{v}_1 t-\tilde{\alpha}_1^2}} S_{n-1}(\tilde{r})\tilde{\phi}[(\tilde{\alpha}_1,\tilde{r},0,\ldots)'|t,\mathbf{0}]d\tilde{r} \\ &= \frac{2}{(\tilde{v}_1 t)^{n+2}}\frac{\Gamma\left(\frac{n+4}{2}\right)}{\sqrt{\pi}\Gamma\left(\frac{n-1}{2}\right)}\int_0^{\sqrt{2\tilde{\alpha}_1\tilde{v}_1 t-\tilde{\alpha}_1^2}}(2\tilde{\alpha}_1\tilde{v}_1 t - \tilde{\alpha}_1^2 - \tilde{r}^2)\tilde{r}^{n-2}d\tilde{r} \\ &= \frac{4}{(n^2-1)(\tilde{v}_1 t)^{n+2}}\frac{\Gamma\left(\frac{n+4}{2}\right)}{\sqrt{\pi}\;\Gamma\left(\frac{n-1}{2}\right)}(2\tilde{\alpha}_1\tilde{v}_1 t - \tilde{\alpha}_1^2)^{\frac{n+1}{2}},\end{aligned} \tag{S21}$$

where the integration is over all classes of mutations with identical fitness. Using Mathematica, the i'th moment of this conditional distribution can be evaluated to

$$\mathrm{E}(\tilde{\alpha}_1^i|t,\mathbf{0}) = \int_0^{2\tilde{v}_1 t}\tilde{\alpha}_1^i\;\tilde{\phi}_1(\tilde{\alpha}_1|t,\mathbf{0})d\tilde{\alpha}_1 = \frac{2^i\Gamma(n+3)\Gamma\left(\frac{n+3+2i}{2}\right)}{\Gamma(n+3+i)\Gamma\left(\frac{n+3}{2}\right)}(\tilde{v}_1 t)^i. \tag{S22}$$

By applying the properties of the Gamma function and cancelling, this can be simplified to

$$\mathrm{E}(\tilde{\alpha}_1^i|t,\mathbf{0}) = \begin{cases} (\tilde{v}_1 t)^i \prod_{j=1}^{i/2} \frac{n+1+i+2j}{n+2+2j} & \text{if } i \text{ is even,} \\ (\tilde{v}_1 t)^i \prod_{j=1}^{(i-1)/2} \frac{n+2+i+2j}{n+2+2j} & \text{if } i \text{ is odd.} \end{cases} \tag{S23}$$

In particular,

$$\mathrm{E}(\tilde{\alpha}_1|t,\mathbf{0}) = \tilde{v}_1 t, \tag{S24}$$

$$\mathrm{E}(\tilde{\alpha}_1^2|t,\mathbf{0}) = \frac{n+5}{n+4}(\tilde{v}_1 t)^2. \tag{S25}$$

The moments of the unconditional distribution are given by

$$\begin{aligned} \mathrm{E}(\tilde{\alpha}_1^i|\mathbf{0}) &= \int_0^\infty \tilde{\alpha}_1^i \left(\int_{\tilde{\alpha}_1/(2\tilde{v}_1)}^\infty \tilde{\phi}_1(\tilde{\alpha}_1|t,\mathbf{0}) \tilde{f}(t|\mathbf{0}) dt \right) d\tilde{\alpha}_1 \\ &= \int_0^\infty \tilde{f}(t|\mathbf{0}) \left(\int_0^{2\tilde{v}_1 t} \tilde{\alpha}_1^i \; \tilde{\phi}_1(\tilde{\alpha}_1|t,\mathbf{0}) d\tilde{\alpha}_1 \right) dt. \end{aligned} \tag{S26}$$

The inner integral equals the conditional moment (eq. S22). Since the latter is proportional to t^i, the unconditional moment is simply

$$\mathrm{E}(\tilde{\alpha}_1^i|\mathbf{0}) = \frac{1}{t^i} \mathrm{E}(\tilde{\alpha}_1^i|t,\mathbf{0}) \; \mathrm{E}(t^i|\mathbf{0}). \tag{S27}$$

For the ease of notation, t denotes a random variable as well as its realization. In particular,

$$\mathrm{E}(\tilde{\alpha}_1|\mathbf{0}) = \left(\frac{\gamma}{\eta(n)\tilde{p}_0}\right)^{\frac{1}{n+3}} \Gamma\left(\frac{n+4}{n+3}\right) \tag{S28}$$

$$\mathrm{Var}(\tilde{\alpha}_1|\mathbf{0}) = \mathrm{E}(\tilde{\alpha}_1^2|\mathbf{0}) - (\mathrm{E}(\tilde{\alpha}_1|\mathbf{0}))^2 = \left(\frac{\gamma}{\eta(n)\tilde{p}_0}\right)^{\frac{2}{n+3}} \left[\frac{n+5}{n+4}\Gamma\left(\frac{n+5}{n+3}\right) - \Gamma\left(\frac{n+4}{n+3}\right)^2 \right]. \tag{S29}$$

As a consequence, the coefficient of variation $\sqrt{\mathrm{Var}(\tilde{\alpha}_1|\mathbf{0})}/\mathrm{E}(\tilde{\alpha}_1|\mathbf{0})$ is independent of γ, η and $\tilde{p}_0$. For the isotropic case, equations (16) and (17) in the main text are obtained by using $\tilde{p}_0 = (2\pi)^{-\frac{n}{2}}$ and transforming back to the original scale with $\boldsymbol{\alpha} = \mathbf{Q}^{-1}\tilde{\boldsymbol{\alpha}} = \bar{m}\tilde{\boldsymbol{\alpha}}$.

Distribution of $\tilde{\alpha}_2$. A similar calculation leads to the moments of the distribution of $\tilde{\alpha}_2$, or indeed of any trait under constant stabilizing selection (i.e., orthogonal to the direction of the moving optimum).

The conditional distribution of $\tilde{\alpha}_2$ is given by

$$\begin{aligned}\tilde{\phi}_2(\tilde{\alpha}_2|t,\mathbf{0}) &= \int_0^{\sqrt{(\tilde{v}_1 t)^2-\tilde{\alpha}_2^2}} S_{n-1}(\tilde{r})\tilde{\phi}[(\tilde{v}_1 t+\tilde{r},\tilde{\alpha}_2,0,\ldots)'|t,\mathbf{0}]d\tilde{r} \\ &= \frac{4}{(n^2-1)(\tilde{v}_1 t)^{n+2}}\frac{\Gamma\left(\frac{n+4}{2}\right)}{\sqrt{\pi}\Gamma\left(\frac{n+1}{2}\right)}[(\tilde{v}_1 t)^2-\tilde{\alpha}_2^2]^{\frac{n+1}{2}},\end{aligned} \tag{S30}$$

with moments

$$\begin{aligned}\mathrm{E}(\tilde{\alpha}_2^i|t,\mathbf{0}) &= \frac{[1+(-1)^i]\Gamma\left(\frac{1+i}{2}\right)\Gamma\left(\frac{n+4}{2}\right)}{2\sqrt{\pi}\;\Gamma\left(\frac{n+4+i}{2}\right)}(\tilde{v}_1 t)^i \\ &= \begin{cases}\prod_{j=1}^{i/2}\frac{(2j-1)}{(n+2+2j)}(\tilde{v}_1 t)^i & \text{if } i \text{ is even,} \\ 0 & \text{if } i \text{ is odd.}\end{cases}\end{aligned} \tag{S31}$$

The unconditional moments are again given by

$$\mathrm{E}(\tilde{\alpha}_2^i|\mathbf{0}) = \frac{1}{t^i}\mathrm{E}(\tilde{\alpha}_2^i|t,\mathbf{0})\;\mathrm{E}(t^i,\mathbf{0}). \tag{S32}$$

In particular, the variance of $\tilde{\alpha}_2$ is

$$\mathrm{Var}(\tilde{\alpha}_2|\mathbf{0}) = \mathrm{E}(\tilde{\alpha}_2^2|\mathbf{0}) = \frac{1}{n+4}\left(\frac{\gamma}{\eta(n)\tilde{p}_0}\right)^{\frac{2}{n+3}}\Gamma\left(\frac{n+5}{n+3}\right). \tag{S33}$$

Furthermore, due to symmetry, all pairs of components of $\tilde{\boldsymbol{\alpha}}$ are uncorrelated (though not independent). Hence, the covariance matrix of $\tilde{\boldsymbol{\alpha}}$ is a diagonal matrix with its first entry given by the right-hand side of equation (S29) and all others by the right-hand side of equation (S33). Furthermore, the covariance matrix in the untransformed space can be obtained from the back-transformation

$$\mathbf{Cov}(\boldsymbol{\alpha}|\mathbf{0}) = \mathbf{Q}^{-1}\mathbf{Cov}(\tilde{\boldsymbol{\alpha}}|\mathbf{0})(\mathbf{Q}^{-1})'. \tag{S34}$$

For the special case of selectional correlation discussed in the main text, one finds

that, for $n = 2$

$$\mathbf{Cov}(\boldsymbol{\alpha}|\mathbf{0}) = \frac{1}{\sqrt{1-\rho_\Sigma^2}} \begin{pmatrix} (1-\rho_\Sigma^2)\mathrm{Var}(\tilde{\alpha}_1|\mathbf{0}) + \rho_\Sigma^2 \mathrm{Var}(\tilde{\alpha}_2|\mathbf{0}) & \rho_\Sigma \mathrm{Var}(\tilde{\alpha}_2|\mathbf{0}) \\ \rho_\Sigma \mathrm{Var}(\tilde{\alpha}_2|\mathbf{0}) & \mathrm{Var}(\tilde{\alpha}_2|\mathbf{0}) \end{pmatrix} \tag{S35}$$

and, hence,

$$\rho_\alpha = \mathrm{Cor}(\alpha_1, \alpha_2|\mathbf{0}) = \rho_\Sigma \sqrt{\frac{\mathrm{Var}(\alpha_2|\mathbf{0})}{\mathrm{Var}(\alpha_1|\mathbf{0})}} \approx \rho_\Sigma \sqrt{(1-\rho_\Sigma^2)0.77 + \rho_\Sigma^2} \approx \rho_\Sigma. \tag{S36}$$

In particular, ρ_α (for the first step and in the environmentally-limited regime) is independent of γ.

Distribution of $\|\tilde{\boldsymbol{\alpha}}\|$. As shown by equation (S19), the distribution of adaptive substitutions depends only on the first component $\tilde{\alpha}_1$ and the total step size $\|\tilde{\boldsymbol{\alpha}}\|$ (i.e., of the Euclidean norm of $\tilde{\boldsymbol{\alpha}}$). To characterize the distribution of $\|\tilde{\boldsymbol{\alpha}}\|$, we again start with the conditional distribution, which can be written as

$$\tilde{\phi}_{\mathrm{norm}}(\|\tilde{\boldsymbol{\alpha}}\| \mid t, \mathbf{0}) = \int_{\frac{\|\tilde{\boldsymbol{\alpha}}\|^2}{2\tilde{v}_1 t}}^{\|\tilde{\boldsymbol{\alpha}}\|} S_{n-1}\left(\sqrt{\|\tilde{\boldsymbol{\alpha}}\|^2 - \tilde{\alpha}_1^2}\right) \tilde{\phi}\left[\left((\tilde{\alpha}_1, \sqrt{\|\tilde{\boldsymbol{\alpha}}\|^2 - \tilde{\alpha}_1^2}, 0, \dots\right)' \mid t, \mathbf{0}\right] \frac{\|\tilde{\boldsymbol{\alpha}}\|}{\sqrt{\|\tilde{\boldsymbol{\alpha}}\|^2 - \tilde{\alpha}_1^2}}\, d\tilde{\alpha}_1. \tag{S37}$$

Here, the last term arises because the integral is calculated along a line in the $(\tilde{\alpha}_1, \tilde{\alpha}_2)$ space, where $\tilde{\alpha}_2 = \sqrt{\|\tilde{\boldsymbol{\alpha}}\|^2 - \tilde{\alpha}_1^2}$. After some rearrangements and using Mathematica, the moments of this distribution can be evaluated to yield

$$\mathrm{E}(\|\tilde{\boldsymbol{\alpha}}\|^i \mid t, \mathbf{0}) = \frac{2^{n+1+i}\Gamma\left(\frac{n+4}{2}\right)\Gamma\left(\frac{n+1+i}{2}\right)}{(n+2+i)\sqrt{\pi}\,\Gamma\left(\frac{2n+2+i}{2}\right)}(\tilde{v}_1 t)^i, \tag{S38}$$

and the unconditional moments are again given by

$$\mathrm{E}(\|\tilde{\boldsymbol{\alpha}}\|^i|\mathbf{0}) = \frac{1}{t^i}\mathrm{E}(\|\tilde{\boldsymbol{\alpha}}\|^i \mid t, \mathbf{0})\ \mathrm{E}(t^i|\mathbf{0}). \tag{S39}$$

Again, the coefficient of variation is independent of γ, η and $\tilde{p}_0$.

Distribution of $\sqrt{\sum_{j=2}^{n}\tilde{\alpha}_j^2}$. We might also be interested in how much deviation from the optimum an adaptive substitution incurs in the traits under constant selection (this can be seen as the "cost" for following the moving optimum). Let this deviation be denoted by $\tilde{\epsilon} = \sqrt{\sum_{j=2}^{n}\tilde{\alpha}_j^2} = \sqrt{\|\tilde{\boldsymbol{\alpha}}\|^2 - \tilde{\alpha}_1^2}$. The conditional distribution of $\tilde{\epsilon}$ is

$$\begin{aligned}\tilde{\phi}_\epsilon(\epsilon|t,\mathbf{0}) &= \int_{\tilde{v}_1 t-\sqrt{(\tilde{v}_1 t)^2-\tilde{\epsilon}^2}}^{\tilde{v}_1 t+\sqrt{(\tilde{v}_1 t)^2-\tilde{\epsilon}^2}} S_{n-1}(\tilde{\epsilon})\tilde{\phi}[\tilde{\alpha}_1,\tilde{\epsilon},0,\ldots)'|t,\mathbf{0}]d\tilde{\alpha}_1 \\ &= \frac{8\ \Gamma\left(\frac{n+4}{2}\right)\epsilon^{n-2}\left((\tilde{v}_1 t)^2-\epsilon^2\right)^{3/2}}{3(\tilde{v}_1 t)^{n+2}\sqrt{\pi}\ \Gamma\left(\frac{n-1}{2}\right)}.\end{aligned} \tag{S40}$$

Its moments are

$$\mathrm{E}(\tilde{\epsilon}^i|t,\mathbf{0}) = \frac{\Gamma\left(\frac{n+4}{2}\right)\Gamma\left(\frac{n-1+i}{2}\right)}{\Gamma\left(\frac{n-1}{2}\right)\Gamma\left(\frac{n+4+i}{2}\right)}(\tilde{v}_1 t)^i, \tag{S41}$$

and the unconditional moments are again $\mathrm{E}(\tilde{\epsilon}^i|\mathbf{0}) = t^{-i}\mathrm{E}(\tilde{\epsilon}^i|t,\mathbf{0})\mathrm{E}(t^i|\mathbf{0})$, with the coefficient of variation independent of γ, η and $\tilde{p}_0$. In addition, we can calculate the covariance between $\tilde{\alpha}_1$ and $\tilde{\epsilon}$. We start with the conditional expectation of the product $\tilde{\alpha}_1\tilde{\epsilon}$,

$$\begin{aligned}\mathrm{E}(\tilde{\alpha}_1\tilde{\epsilon}|t,\mathbf{0}) &= \int_0^{2\tilde{v}_1 t}\int_0^{\sqrt{2\tilde{\alpha}_1\tilde{v}_1 t-\alpha_1^2}} \tilde{\alpha}_1\tilde{\epsilon}S_{n-1}(\tilde{\epsilon})\tilde{\phi}((\tilde{\alpha}_1,\tilde{\epsilon},0,\ldots)|t,\mathbf{0})d\tilde{\epsilon}d\tilde{\alpha}_1 \\ &= \frac{(n+2)\Gamma\left(\frac{n+2}{2}\right)^2}{n\Gamma\left(\frac{n-1}{2}\right)\Gamma\left(\frac{n+5}{2}\right)}(\tilde{v}_1 t)^2.\end{aligned} \tag{S42}$$

The unconditional expectation is $\mathrm{E}(\tilde{\alpha}_1\tilde{\epsilon}|\mathbf{0}) = \frac{1}{t^2}\mathrm{E}(\tilde{\alpha}_1\tilde{\epsilon}|\mathbf{0})\mathrm{E}(t^2|\mathbf{0})$, and the covariance is given by

$$\mathrm{Cov}(\tilde{\alpha}_1,\tilde{\epsilon}|\mathbf{0}) = \mathrm{E}(\tilde{\alpha}_1\tilde{\epsilon}|\mathbf{0}) - \mathrm{E}(\tilde{\alpha}_1|\mathbf{0})\mathrm{E}(\tilde{\epsilon}|\mathbf{0}). \tag{S43}$$

Furthermore, it is easy to show that the coefficient of correlation

$$\rho(\tilde{\alpha}_1,\tilde{\epsilon}|\mathbf{0}) = \frac{\mathrm{Cov}(\tilde{\alpha}_1,\tilde{\epsilon}|\mathbf{0})}{\sqrt{\mathrm{Var}(\tilde{\alpha}_1|\mathbf{0})\mathrm{Var}(\tilde{\epsilon}|\mathbf{0})}} \tag{S44}$$

is independent of γ, η and $\tilde{p}_0$.

An illustration of some of these results is give in Figure S**4**_1. Note that all quantities depend on γ only through the moments of the waiting-time distribution (eq. S18),

whose power-law form leads to the linear relationship in double-log plots, with the slope for the i'th moment given by the exponent $i/(n+3)$. Since this slope decreases with n (ultimately a geometric consequence of equation S13), the lines in Figure S**4**_1 cross at high values of γ. Biologically, this means that, while at a given distance to the optimum, the proportion of beneficial mutations is smaller in complex organisms, the same proportion increases faster as the distance to the optimum increases. As a consequence, at very high values of γ, the relationship between mean step size and n is reversed, with more complex organisms being predicted to evolve in smaller steps. However, this effect only sets in if the environment changes so fast that realistic populations cannot follow anyway and go extinct right away.

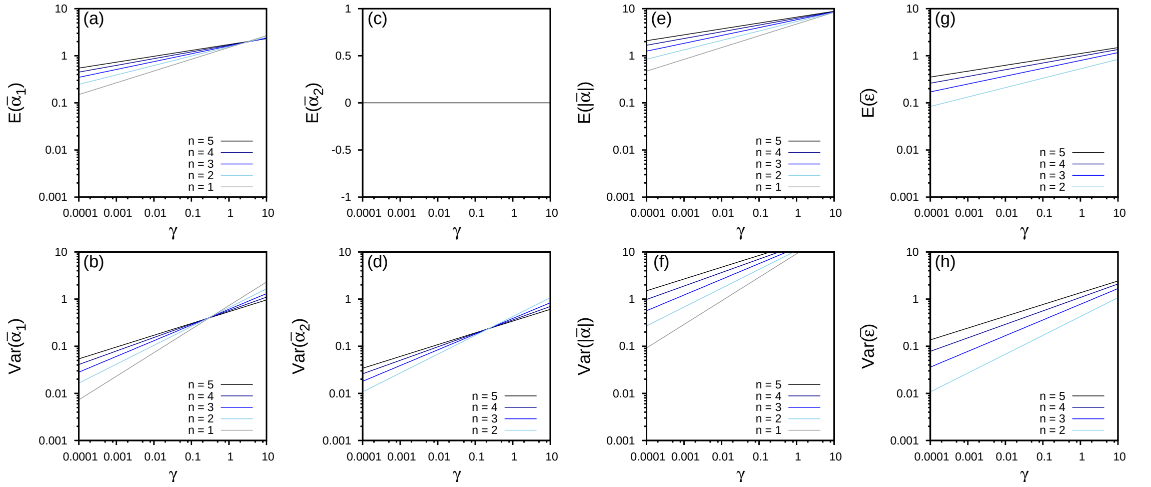

Figure S4_1: Dependence of various components of the first adaptive step $\tilde{\boldsymbol{\alpha}}$ in the transformed phenotype space, as a function of the scaled rate of environmental change γ for various numbers of traits n, assuming a uniform distribution of new mutations. (a,b) Expectation and variance of the step size in the direction of the moving optimum, $\tilde{\alpha}_1$ (eq. S28 and S29); (c, d) expectation and variance of step size in an direction orthogonal to the moving optimum, $\tilde{\alpha}_1$ (eq. S33); (e, f) expectation and variance of the total step size (Euclidean norm), $\|\tilde{\boldsymbol{\alpha}}\|$ (based on eq. S39); (g, h) expectation and variance of the total deviation from the optimum in traits under constant stabilizing selection, $\tilde{\epsilon} = \sqrt{\sum_{j=2}^{n} \tilde{\alpha}_j^2}$ (based on eq. S41).

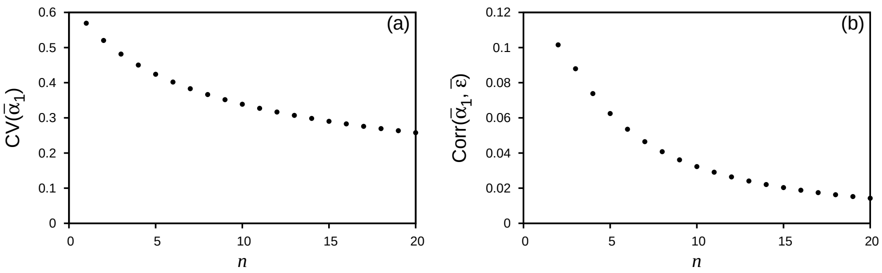

Figure S4_2: (a) Coefficient of variation of the size of the first step in the direction of the moving optimum, $\sqrt{\mathrm{Var}(\tilde{\alpha}_1)}/\mathrm{E}(\tilde{\alpha}_1)$. (b) Correlation coefficient between the size of the first step in the direction of the moving optimum and its total deviation from the optimum in traits under constant stabilizing selection, $\rho(\tilde{\alpha}_1, \tilde{\epsilon})$ (eq. S44). Both quantities depend only on the number of traits, n. Results are valid in the transformed phenotype space and assume a uniform distribution of new mutations.

Supporting Information 5: Supplementary Figures

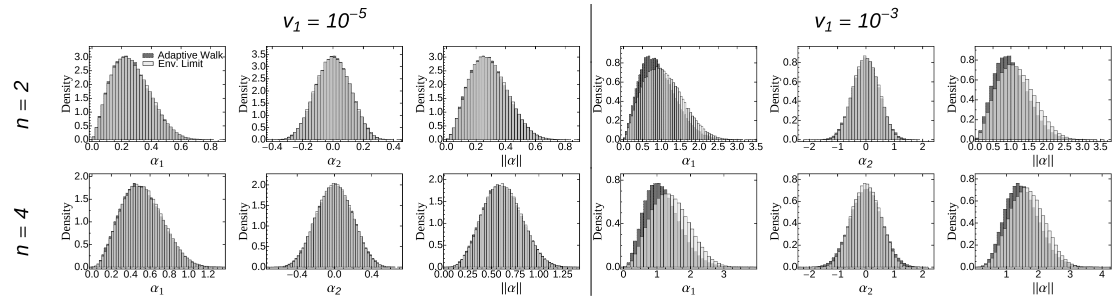

Figure S5_1: Distribution of the size of the first adaptive substitution, for two (top row) and four (bottom row) traits and two different rates of environmental change v_1. For each rate, columns display the distribution of step sizes in direction of the moving optimum (α_1, left column), the distribution of step sizes for the trait under stabilizing selection (α_2, central column) and the distribution of the total step sizes ($\|\boldsymbol{\alpha}\|$, right column). Results are shown for adaptive-walk simulations (dark bins) assuming a normally-distributed distribution of new mutations and for the approximation eq. S19, which is based on a uniform distribution of new mutations (light bins). Note that the scales of the axes vary between plots. Parameters: $\Theta = 1, \sigma^2 = 10, \rho_\Sigma = 0, m^2 = 1, \rho_M = 0$. Scaled rate of environmental change $\gamma = 10 \cdot v_1$.

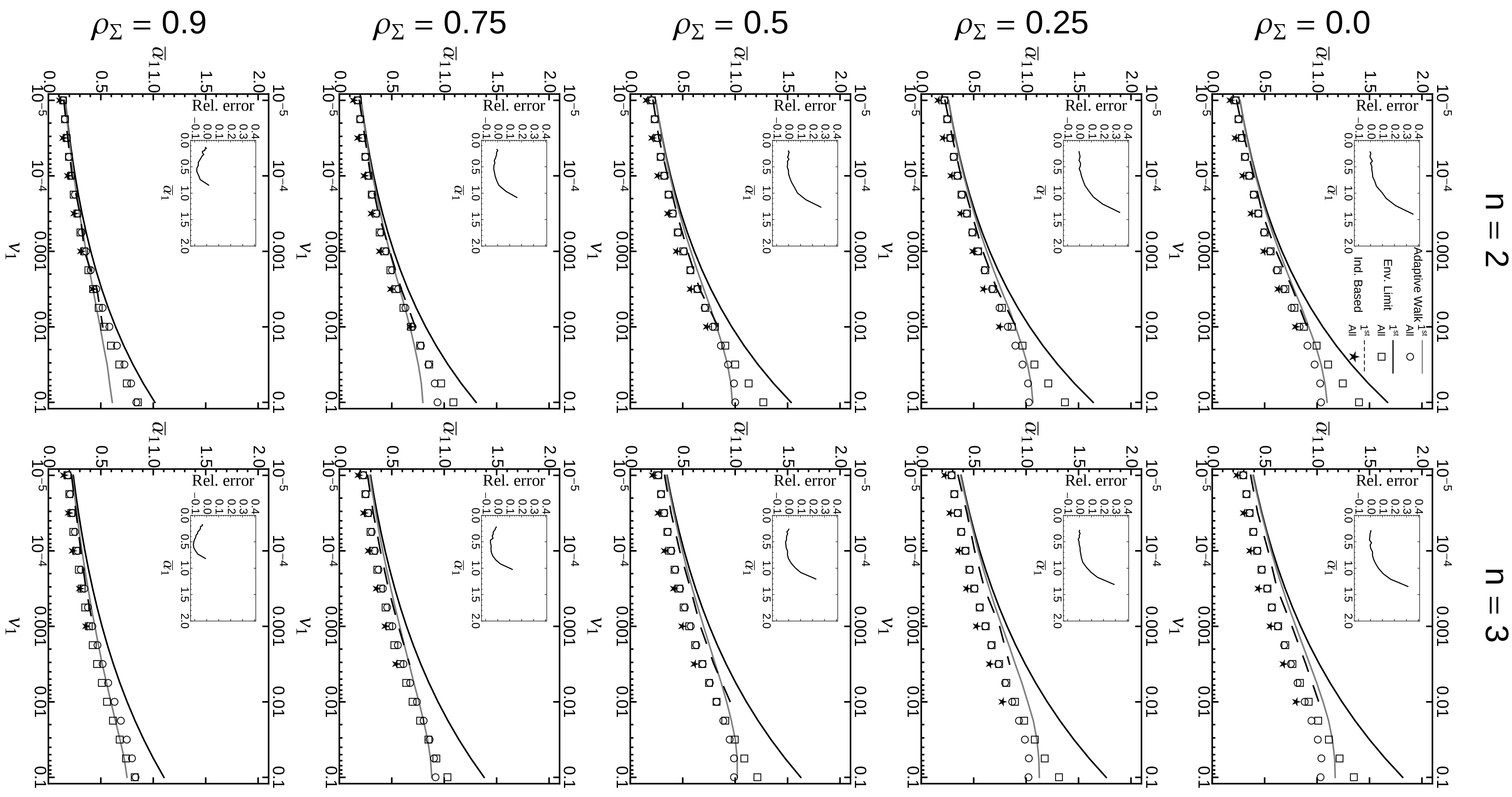

Figure S5_2: Mean size $\bar{\alpha}_1$ of adaptive substitutions in the direction of the moving optimum, for the first step (lines) and over the entire adaptive walk (symbols), as a function of the rate of environmental change v_1, for $n = 2$ and 3 traits and different values of selectional correlation ρ_Σ. "Adaptive walk" refers to adaptive-walk simulations with a normal distribution of new mutations, "Env. limit" to adaptive-walk simulations assuming a uniform distribution of new mutations (eq. S19), and "Ind. based" to individual-based simulations (with normally distributed new mutations). Also shown is the relative error over all steps incurred by "Env. limit" relative to "Adaptive walk". Parameters: $\Theta = 1, \sigma^2 = 10, m^2 = 1, \rho_M = 0.0$.

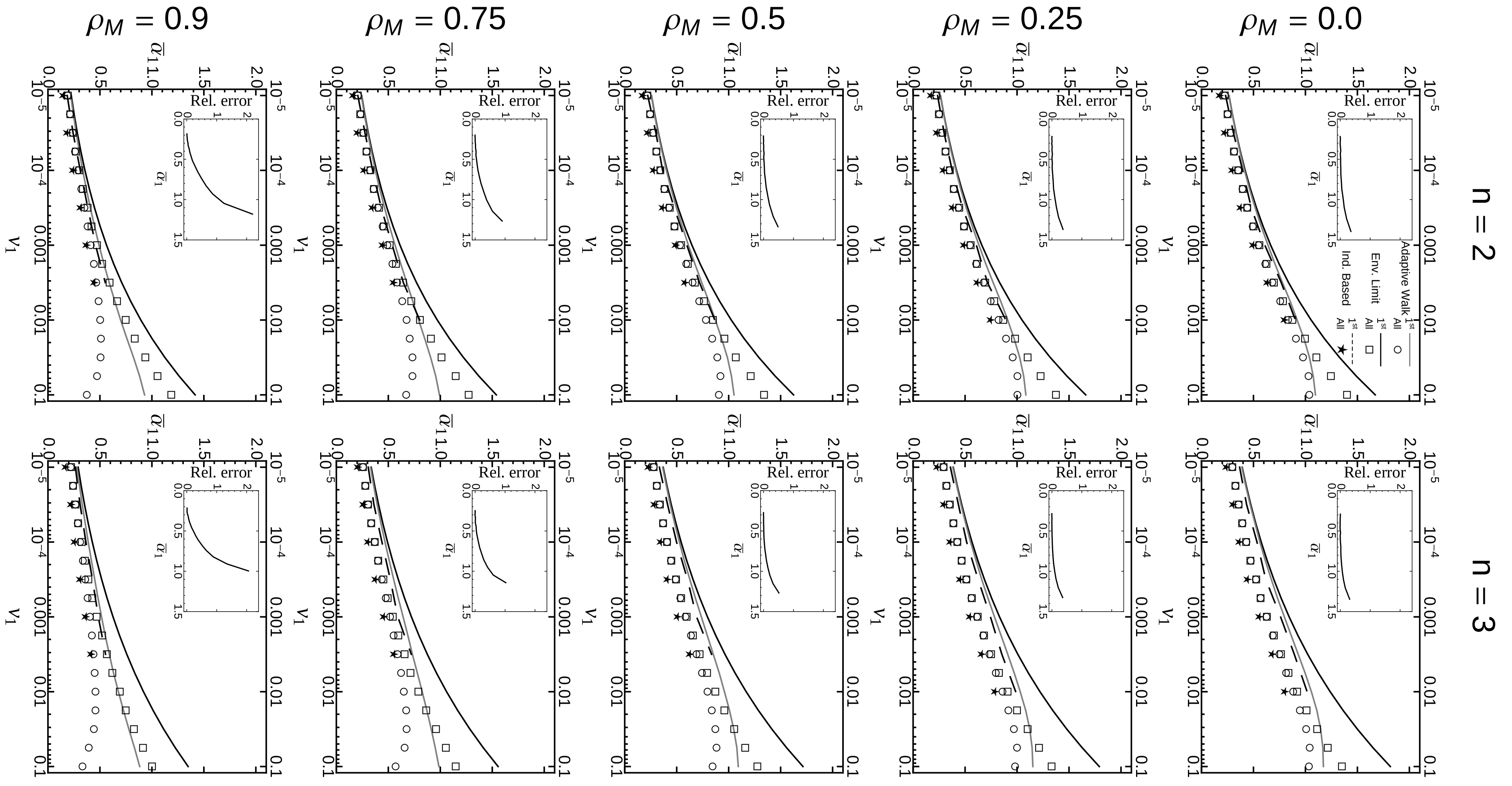

Figure S5_3: Mean size $\bar{\alpha}_1$ of adaptive substitutions in the direction of the moving optimum, for the first step (lines) and over the entire adaptive walk (symbols), as a function of the rate of environmental change v_1, for $n = 2$ and 3 traits and different values of mutational correlation ρ_M. "Adaptive walk" refers to adaptive-walk simulations with a normal distribution of new mutations, "Env. limit" to adaptive-walk simulations assuming a uniform distribution of new mutations (eq. S19), and "Ind. based" to individual-based simulations (with normally distributed new mutations). Also shown is the relative error over all steps incurred by "Env. limit" relative to "Adaptive walk". Parameters: $\Theta = 1, \sigma^2 = 10, \rho_\Sigma = 0.0, m^2 = 1$.

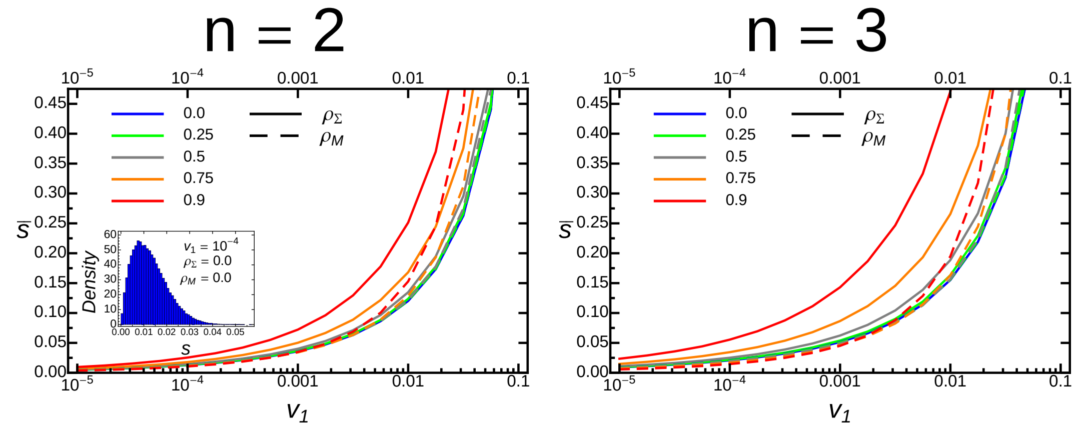

Figure S5_4: Mean selection coefficient of adaptive substitutions as a function of the rate of environmental change for various strengths of mutational (dashed lines) and selectional (solid lines) correlations ρ_M and ρ_Σ. The inset gives a representative distribution of the selection coefficient for the isotropic case ($\rho_M = \rho_\Sigma = 0.0$) and $v_1 = 10^{-4}$. Note that with increasing v_1 the distribution becomes more asymmetric. Parameters: $\Theta = 1$.

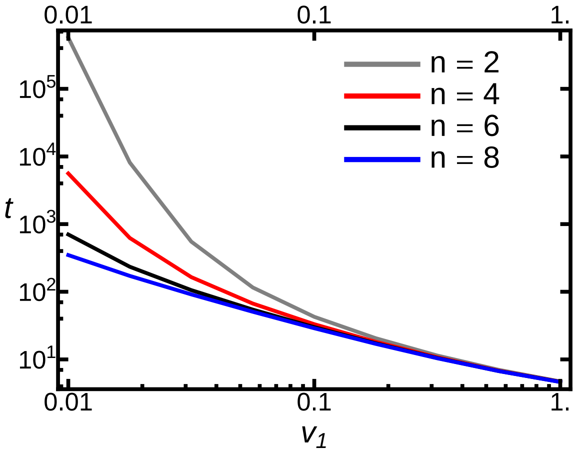

Figure S5_5: The mean time to extinction for different levels of phenotypic complexity n as a function of the rate of environmental change. The mean extinction time was calculated based on $100,000$ adaptive-walk simulations, where populations were considered extinct when the mean fitness dropped below 0.5. Simulations that persisted for more than $1,000,000$ generations were aborted for performance reasons. Parameter values are $\Theta = 1, \sigma^2 = 10, \rho_\Sigma = 0.0, m^2 = 1$; the scaled rate of environmental change $\gamma = 10v_1$.

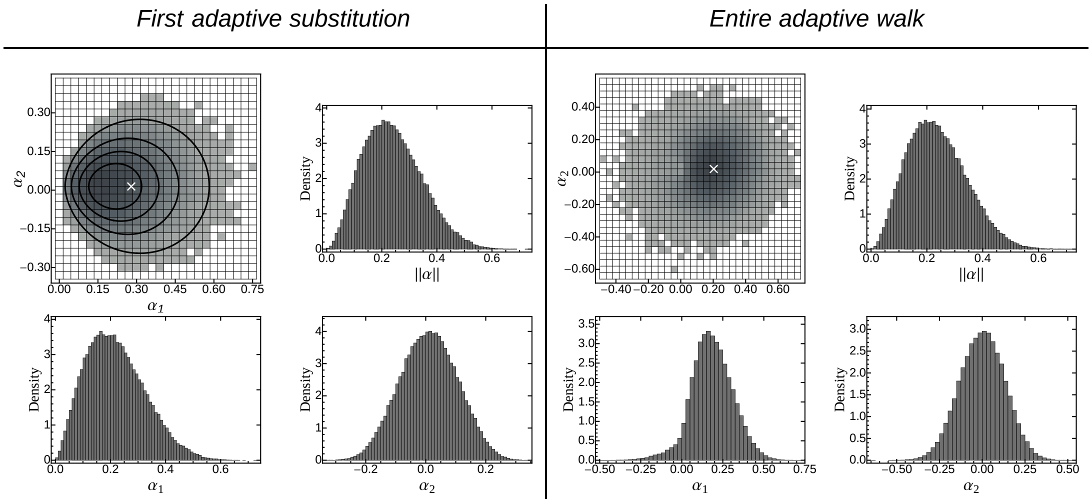

Figure S5_6: The multivariate distribution of the first adaptive substitution (left) and over the entire adaptive walk (right) for $n = 2$ traits, when the optimum moves slowly in the direction of the first trait and the effects of new mutations are strongly correlated ($\rho_M = 0.9$). In the top-left figures on each side, shades of grey indicate the frequency of a given step size in adaptive-walk simulations with normally-distributed mutational effects (with dark grey corresponding to high frequency), with the white cross showing the observed mean. The contour lines on the left represent the probability density intervals predicted for a uniform distribution of new mutations (environmentally-limited regime, eq. S19; highest probability density intervals for $0.25, 0.5, 0.75, 0.95$ from inside out). Histograms show the marginal distribution of the first and second trait, α_1 and α_2, and the distribution of the total step size $\|\boldsymbol{\alpha}\|$. Parameter values are $v_1 = 10^{-5}, \Theta = 1, \sigma^2 = 10, \rho_\Sigma = 0.0, m^2 = 1$; the scaled rate of environmental change $\gamma = 10^{-4}$.

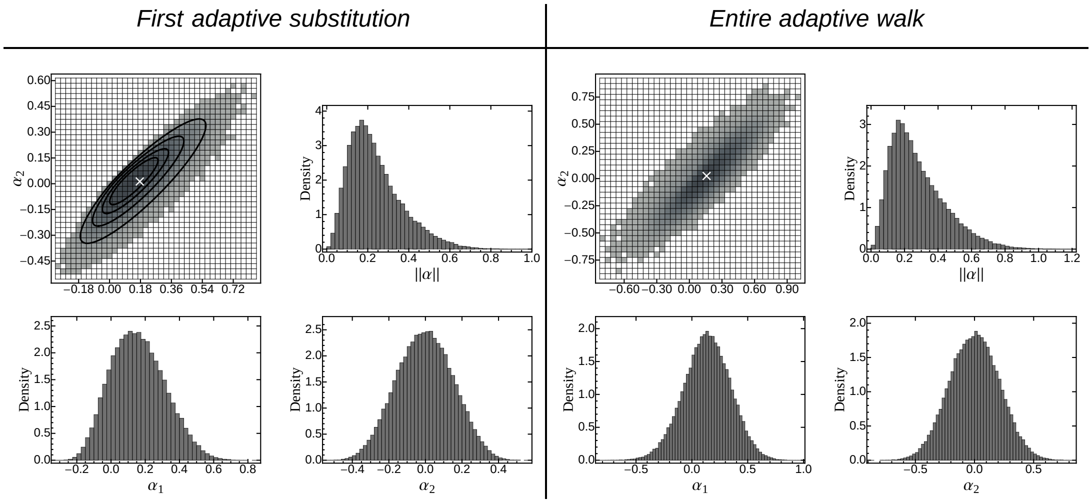

Figure S5_7: The multivariate distribution of the first adaptive substitution (left) and over the entire adaptive walk (right) for $n = 2$ traits, when the optimum moves slowly in the direction of the first trait and selection is strongly correlated ($\rho_\Sigma = 0.9$). In the top-left figures on each side, shades of grey indicate the frequency of a given step size in adaptive-walk simulations with normally-distributed mutational effects (with dark grey corresponding to high frequency), with the white cross showing the observed mean. The contour lines on the left represent the probability density intervals predicted for a uniform distribution of new mutations (environmentally-limited regime, eq. S19; highest probability density intervals for $0.25, 0.5, 0.75, 0.95$ from inside out). Histograms show the marginal distribution of the first and second trait, α_1 and α_2, and the distribution of the total step size $\|\boldsymbol{\alpha}\|$. Parameter values are $v_1 = 10^{-5}, \Theta = 1, \sigma^2 = 10, m^2 = 1, \rho_M = 0$; the scaled rate of environmental change $\gamma = 10^{-4}$.

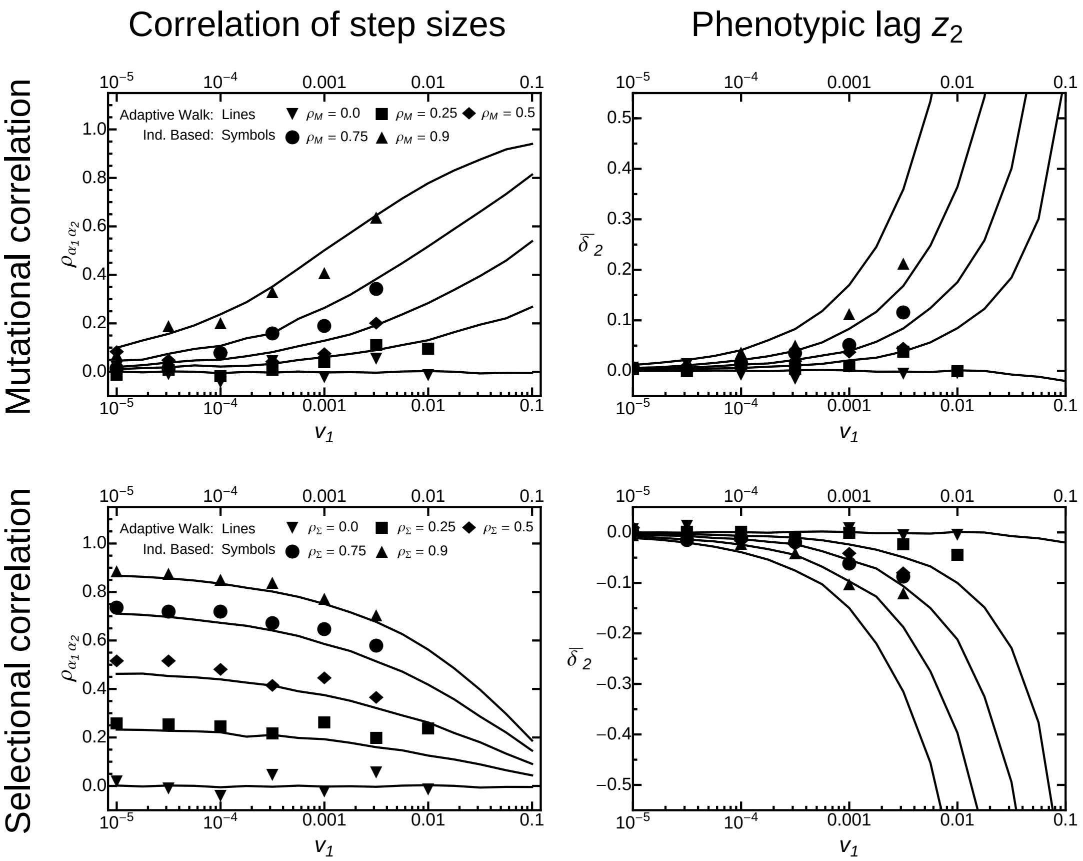

Figure S5_8: The impact of mutational and selectional correlations on the distribution of adaptive substitutions for $n = 3$ traits. For details, see Fig. 5 of the main text.

.

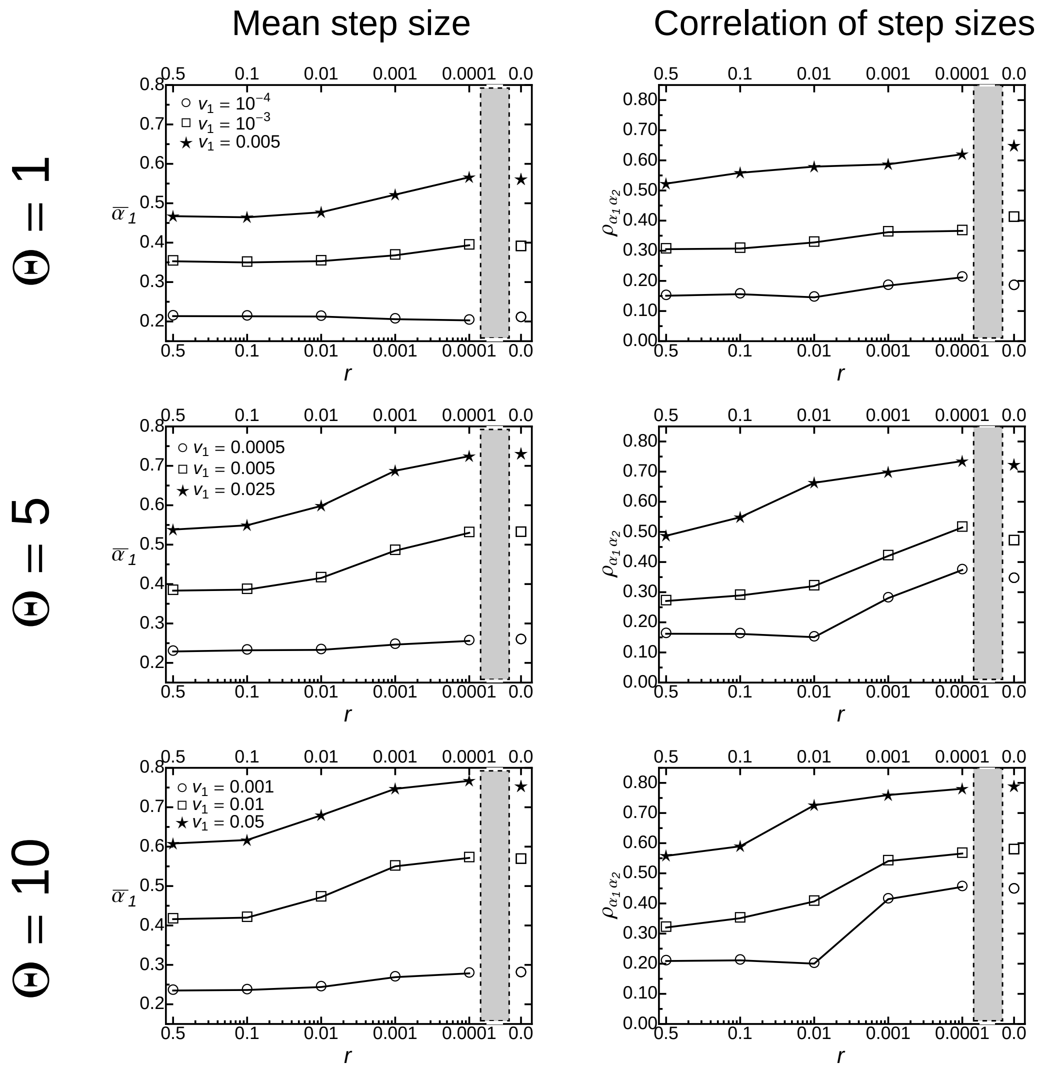

Figure S5_9: The effects of linkage and interference between co-segregating alleles on the mean step size in direction of the moving optimum α_1 (left) and the correlation between adaptive substitutions ρ_{α_1,α_2} (right) under strong mutational correlations $\rho_M = 0.9$. The plots are based on 5000 replicated individual-based simulations. The population-wide mutation rate Θ was varied by increasing the per-locus mutation rate μ. Rates of environmental change v_1 were chosen such that the same three values of γ (i.e., the scaled rate of environmental change; see above; $\gamma(\circ) = 0.0035, \gamma(\square) = 0.035, \gamma(\bigstar) = 0.17$) applied for each Θ. Other parameters: $K = 1000, L = 10, \sigma^2 = 10, \rho_\Sigma = 0.0, m^2 = 1$.

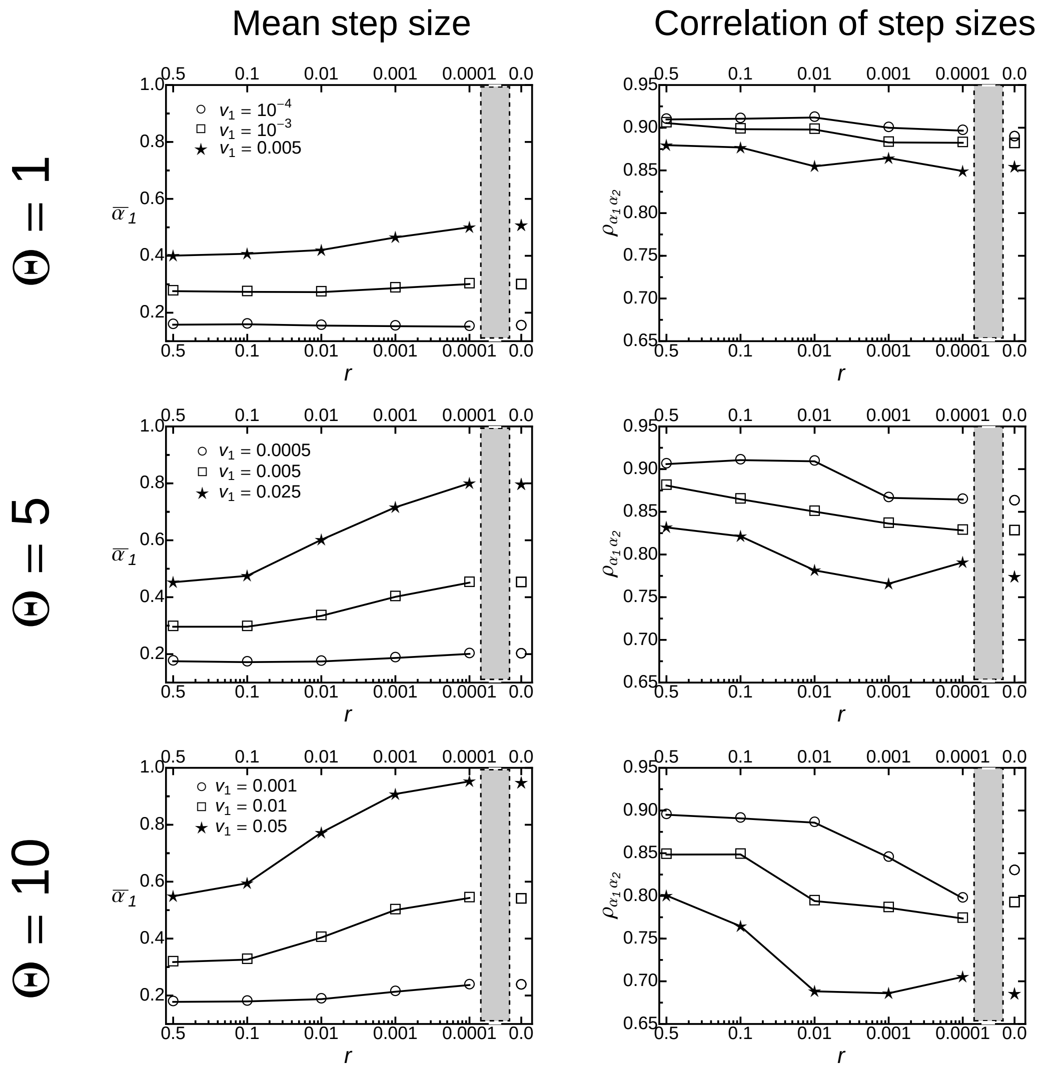

Figure S5_10: The effects of linkage and interference between co-segregating alleles on the mean step size in direction of the moving optimum α_1 (left) and the correlation between adaptive substitutions ρ_{α_1,α_2} (right) under strong selectional correlations $\rho_\Sigma = 0.9$. The plots are based on 5000 replicated individual-based simulations. The population-wide mutation rate Θ was varied by increasing the per-locus mutation rate μ. Rates of environmental change v_1 were chosen such that the same three values of γ (i.e., the scaled rate of environmental change; $\gamma(\circ) = 0.00066, \gamma(\square) = 0.0066, \gamma(\bigstar) = 0.033$) applied for each Θ. Other parameters: $K = 1000, L = 10, \sigma^2 = 10, m^2 = 1, \rho_M = 0.0$.

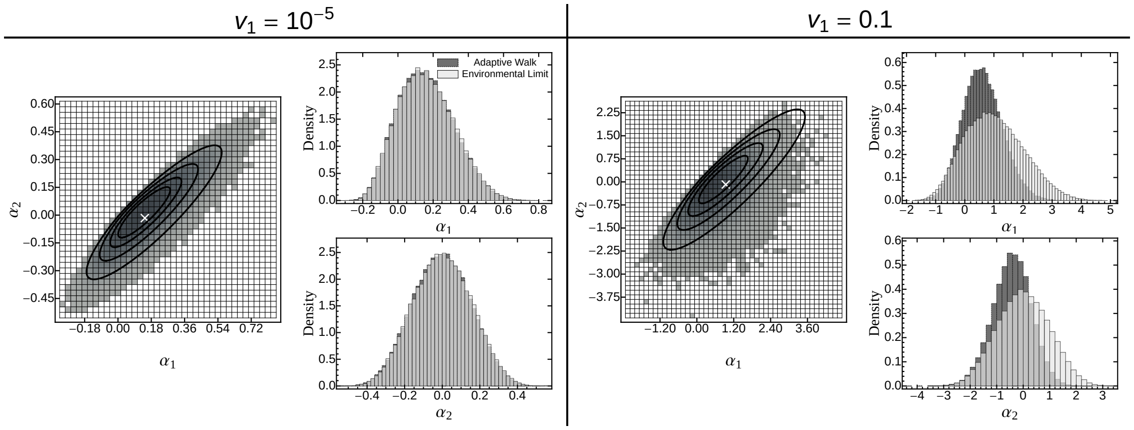

Figure S5_11: The multivariate distribution of the first adaptive substitution in adaptive-walk simulations with strong selectional correlation ($\rho_\Sigma = 0.9$), illustrating the "diving-kite effect" (the negative bias in the α_2-direction) present for fast ($v_1 = 0.1$) but not for slow ($v_1 = 10^{-5}$) environmental change. The left-hand plot on each side is as in Figure S**5**_7, with shades of grey illustrating the distribution in adaptive-walk simulations with normally-distributed new mutations, and contour lines showing the prediction for a uniform distribution of new mutations (environmentally-limited regime, eq. S19). The histograms compare the marginal distributions of both traits for the two distributions of new mutations. Other parameters are identical to those in Figure S**5**_7.

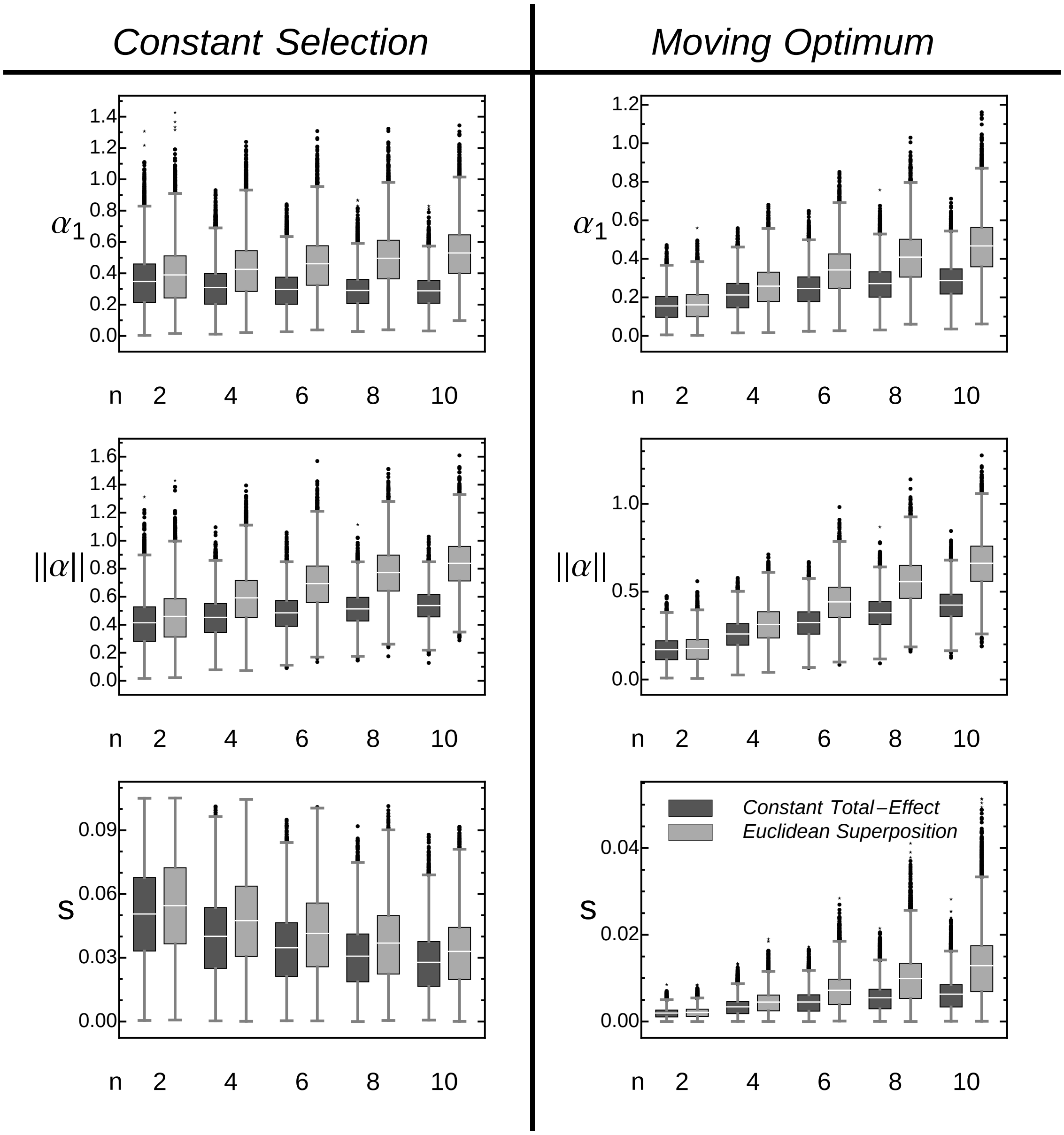

Figure S5_12: Comparison of the first adaptive substitution under the constant total-effect (darker grey) and the Euclidean superposition model (lighter grey) for Fisher's geometric model with constant selection (left) and a moving optimum (right). The boxplots are based on 10000 replicated adaptive-walk simulations and show the distribution of step sizes in direction of the optimum α_1 (top row), the distribution of total step sizes $\|\boldsymbol{\alpha}\|$ and the distribution of the selection coefficient s of adaptive substitutions. Whiskers extend to maximally 1.5 times the size of the box. Horizontal white bars indicate the mean. In the constant selection case, the population started at an initial distance of 1 from the optimum. In the moving-optimum model, the rate of environmental change was $v_1 = 10^{-5}$. The variance of mutational effects was $m^2 = 0.1$ in the Euclidean superposition model and $0.1/\left(\sqrt{2}(\Gamma\left[(n+1)/2\right]/\Gamma\left[n/2\right])\right)$ in the constant total-effect model (here, the denominator is the expected mean of the norm of a multinormal distribution with covariance matrix $\mathbf{I}$, which is equal to the expectation of a χ-distribution with n degrees of freedom). Note that the constant total-effects model used here differs from the one in Orr (2000), because Orr only considered mutations of a single fixed total effect $\|\boldsymbol{\alpha}\|$ (i.e., his mutations are drawn from the surface of a hypersphere, whereas ours are drawn from a multivariate normal distribution). Other parameters: $\Theta = 1, \sigma^2 = 10, \rho_\Sigma = 0.0, \rho_M = 0.0$.

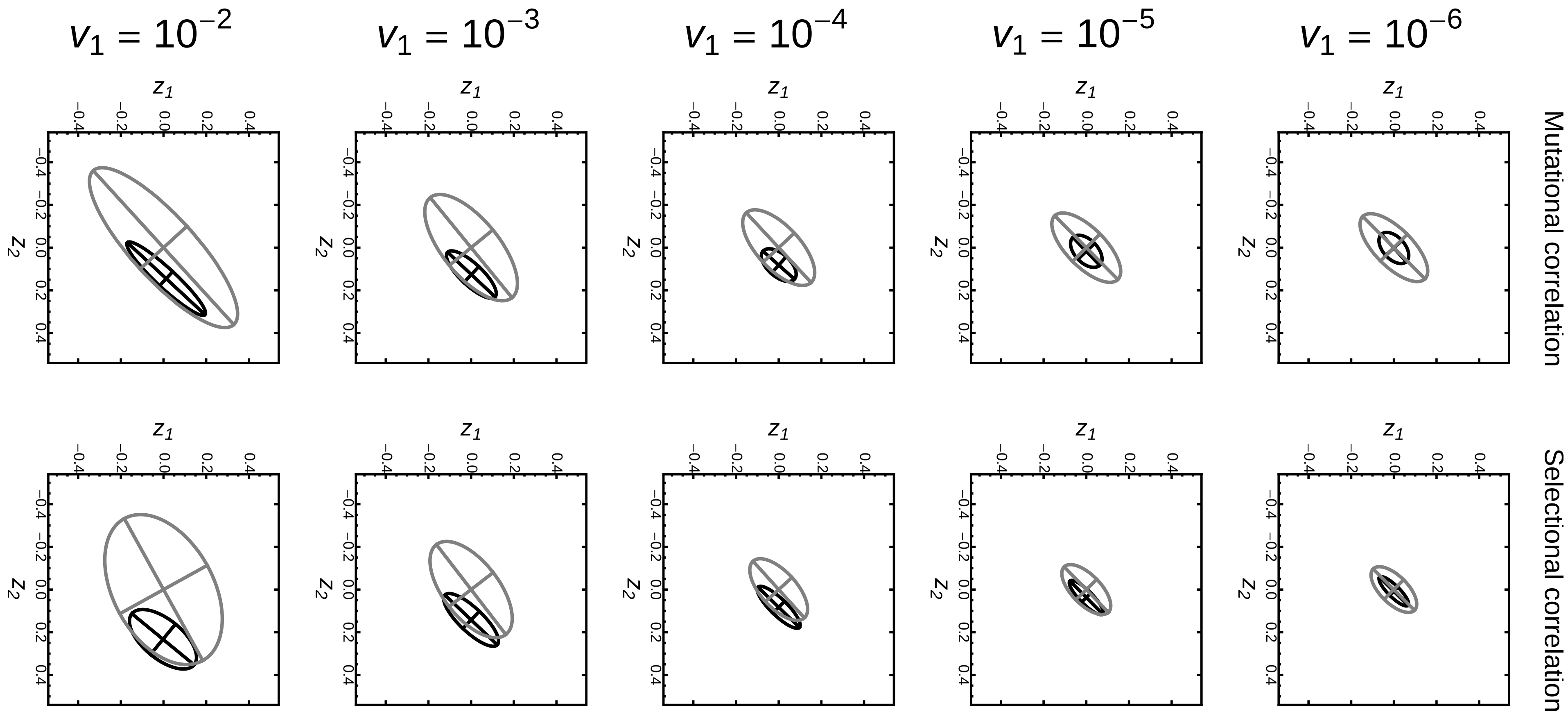

Figure S5_13: The **G** matrix (grey ellipse) and the 90%-confidence ellipse of the distribution of adaptive substitutions (dark ellipse) under strong mutational ($\rho_M = 0.9$) and selectional ($\rho_\Sigma = 0.9$) correlation, for various rates of environmental change (v_1). Results are shown for individual-based simulations of 1000 adaptive substitutions. **G** was calculated as an average over samples taken every 100th generation. Note that **G** increases with v_1. Parameters: $K = 1000$, $L = 50$, $\mu = 5 \times 10^{-5}$, $\sigma^2 = 10$, $m^2 = 0.05$.

References

Gros, P.-A., H. Le Nagard, and O. Tenaillon, 2009. The evolution of epistasis and its links with genetic robustness, complexity and drift in a phenotypic model of adaptation. Genetics 182:277–293.

Hartl, D. L. and C. H. Taubes, 1998. Towards a theory of evolutionary adaptation. Genetics 102-103:525–533.

Kopp, M. and J. Hermisson, 2009. The genetic basis of phenotypic adaptation II: The distribution of adaptive substitutions in the moving optimum model. Genetics 183:1453–1476.

Orr, H. A., 2000. Adaptation and the cost of complexity. Evolution 54:13–20.

Tenaillon, O., O. K. Silander, J.-P. Uzan, and L. Chao, 2007. Quantifying organismal complexity using a population genetic approach. PLoS ONE 2:e217.
